# Ultrasound-based radiomics XGBoost model to assess the risk of central cervical lymph node metastasis in patients with papillary thyroid carcinoma: Individual application of SHAP

**DOI:** 10.3389/fonc.2022.897596

**Published:** 2022-08-26

**Authors:** Yan Shi, Ying Zou, Jihua Liu, Yuanyuan Wang, Yingbin Chen, Fang Sun, Zhi Yang, Guanghe Cui, Xijun Zhu, Xu Cui, Feifei Liu

**Affiliations:** ^1^ Binzhou Medical University Hospital, Binzhou, China; ^2^ First Teaching Hospital of Tianjin University of Traditional Chinese Medicine, Tianjin, China; ^3^ National Clinical Research Center for Chinese Medicine Acupuncture and Moxibustion, Tianjin, China; ^4^ Nanjing No. 1 Hospital, Nanjing, China; ^5^ Peking University People’s Hospital, Beijing, China

**Keywords:** radiomics, lymphatic metastasis, papillary thyroid carcinoma, ultrasound, machine learning

## Abstract

**Objectives:**

A radiomics-based explainable eXtreme Gradient Boosting (XGBoost) model was developed to predict central cervical lymph node metastasis (CCLNM) in patients with papillary thyroid carcinoma (PTC), including positive and negative effects.

**Methods:**

A total of 587 PTC patients admitted at Binzhou Medical University Hospital from 2017 to 2021 were analyzed retrospectively. The patients were randomized into the training and test cohorts with an 8:2 ratio. Radiomics features were extracted from ultrasound images of the primary PTC lesions. The minimum redundancy maximum relevance algorithm and the least absolute shrinkage and selection operator regression were used to select CCLNM positively-related features and radiomics scores were constructed. Clinical features, ultrasound features, and radiomics score were screened out by the Boruta algorithm, and the XGBoost model was constructed from these characteristics. SHapley Additive exPlanations (SHAP) was used for individualized and visualized interpretation. SHAP addressed the cognitive opacity of machine learning models.

**Results:**

Eleven radiomics features were used to calculate the radiomics score. Five critical elements were used to build the XGBoost model: capsular invasion, radiomics score, diameter, age, and calcification. The area under the curve was 91.53% and 90.88% in the training and test cohorts, respectively. SHAP plots showed the influence of each parameter on the XGBoost model, including positive (i.e., capsular invasion, radiomics score, diameter, and calcification) and negative (i.e., age) impacts. The XGBoost model outperformed the radiologist, increasing the AUC by 44%.

**Conclusions:**

The radiomics-based XGBoost model predicted CCLNM in PTC patients. Visual interpretation using SHAP made the model an effective tool for preoperative guidance of clinical procedures, including positive and negative impacts.

## Introduction

Central cervical lymph node metastasis (CCLNM) is a critical factor affecting prognosis and recurrence in papillary thyroid carcinoma (PTC) patients (1). Therefore, preoperative prediction of CCLNM in an accurate and non-invasive manner is important. Ultrasound is the preferred method for evaluating PTC according to American Thyroid Association (ATA) guidelines (2). However, ultrasound shows limitations for assessing central cervical lymph nodes because of the interference from gas in the esophagus and trachea. Ultrasound only detects 20-31% of CCLNM preoperatively and only alters the surgical procedure of 20% of patients (3).

Radiomics mine quantitative image features from medical imaging in a high-throughput manner to improve predictive, diagnostic, and prognostic accuracy (4). Radiomics has shown clinical importance in breast (5), thyroid (6), bladder (7), and colorectal cancers (8). Ultrasound-based radiomics can assess lymph node metastasis in PTC patients to some extent (9, 10). Previous studies were primarily based on radiomics features, and logistic regression analysis was used to construct a nomogram for clinical prediction. The logistic model has good interpretability, and its model coefficient represents the importance of features to the prediction results. However, some variables with a causal relationship with the output variables may not be statistically significant (11). If variables are excluded only from statistical assumptions, available information will be reduced and features of improving prediction ability may be missed.

Machine learning is widely used in the medical field and has a high predictive accuracy (12). However, this model is a complex nonlinear relationship and the limitation of its application in clinical practice is caused by the inexplicability of the model. Several classification algorithms were used to compare diagnostic performance, such as eXtreme Gradient Boosting (XGBoost), deep learning, and transfer learning (6, 13, 14). However, these algorithms all have the “epistemic opacity” problem. The SHapley Additive exPlanation (SHAP) concept was introduced to solve the inexplicability bug (15). SHAP was successfully used to assess mortality in patients with gastrointestinal bleeding (16), prognosis of COVID-19 (17), and mortality in critically ill influenza patients (18).

Here, we constructed a radiomics-based machine learning model based on the ultrasound features of primary PTC lesions. We examined whether SHAP could perform interpretation of CCLNM. The purposes were as follows: to extract the critical features for predicting CCLNM; to establish radiomics-based machine learning model based on key features for CCLNM prediction; and use SHAP to complete the individualized visual interpretation.

## Materials and methods

### Patients

**Figure 1:**
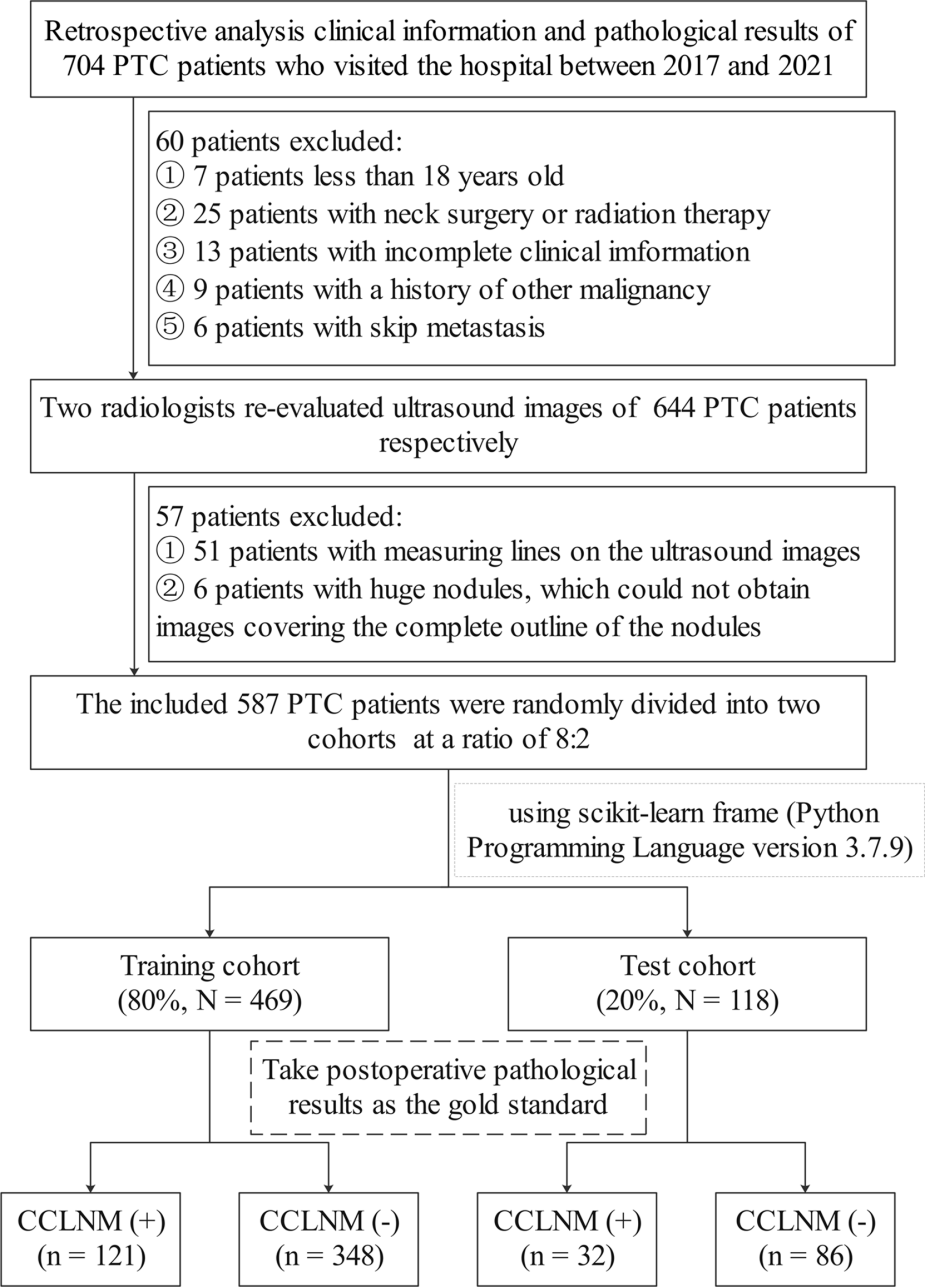
Flowchart of patient selection and group allocation for the study.

The pathological records of 704 PTC patients at Binzhou Medical University Hospital, Shandong, China (2017–2021) were retrospectively analyzed. Exclusion criteria were neck surgery or radiation therapy; history of other malignancy; measuring lines on the ultrasound images; nodules too large to obtain images covering the complete outline of the nodules, even after adjusting the scanning section position; and lacking complete clinical information. All patients were older than 18 years old with complete ultrasound image data. On the other hand, skip metastases are defined as lateral lymph node metastasis without the involvement of CCLNM in PTC. For such patients, although there was no CCLNM, the presence of metastatic lymph nodes in the lateral cervical region may also affect the model we constructed, so we excluded the patients with skip metastasis. Finally, the study included 587 PTC patients. Scikit-learning frame (Python programming language, version 3.7.9) was used to divided the patients into the training and test cohorts at a ratio of 8:2 randomly (**Figure 1**). CCLNM was diagnosed by pathological evaluation. The principles of operation for PTC patients are shown in **Appendix 1**. This study was approved by the institutional ethics committee of Binzhou Medical University Hospital (No. LW-024). Given the retrospective nature of the study, the requirement for written informed consent was waived.

### Ultrasound image acquisition and analysis

Data were scanned with three different Doppler ultrasonic diagnostic apparatuses (**Appendix 2**). The diagnostic criteria of PTC on ultrasound were based on ATA guidelines. Ultrasound image analysis methods are shown in **Appendix 3** and **Appendix Figure 1**. Ultrasound parameters included diameter, location (right lobe, left lobe, or isthmus), composition (mixed or solid), echogenicity (hyper/isoechoic, hypoechoic, or very hypoechoic), shape (wider-than-tall or taller-than-wide), margin (smooth, ill-defined, or lobulated/irregular), calcification (none, macrocalcification, rim calcification, or microcalcification), vascularization, and capsular invasion.

### Radiomics workflow and score

**Figure 2:**
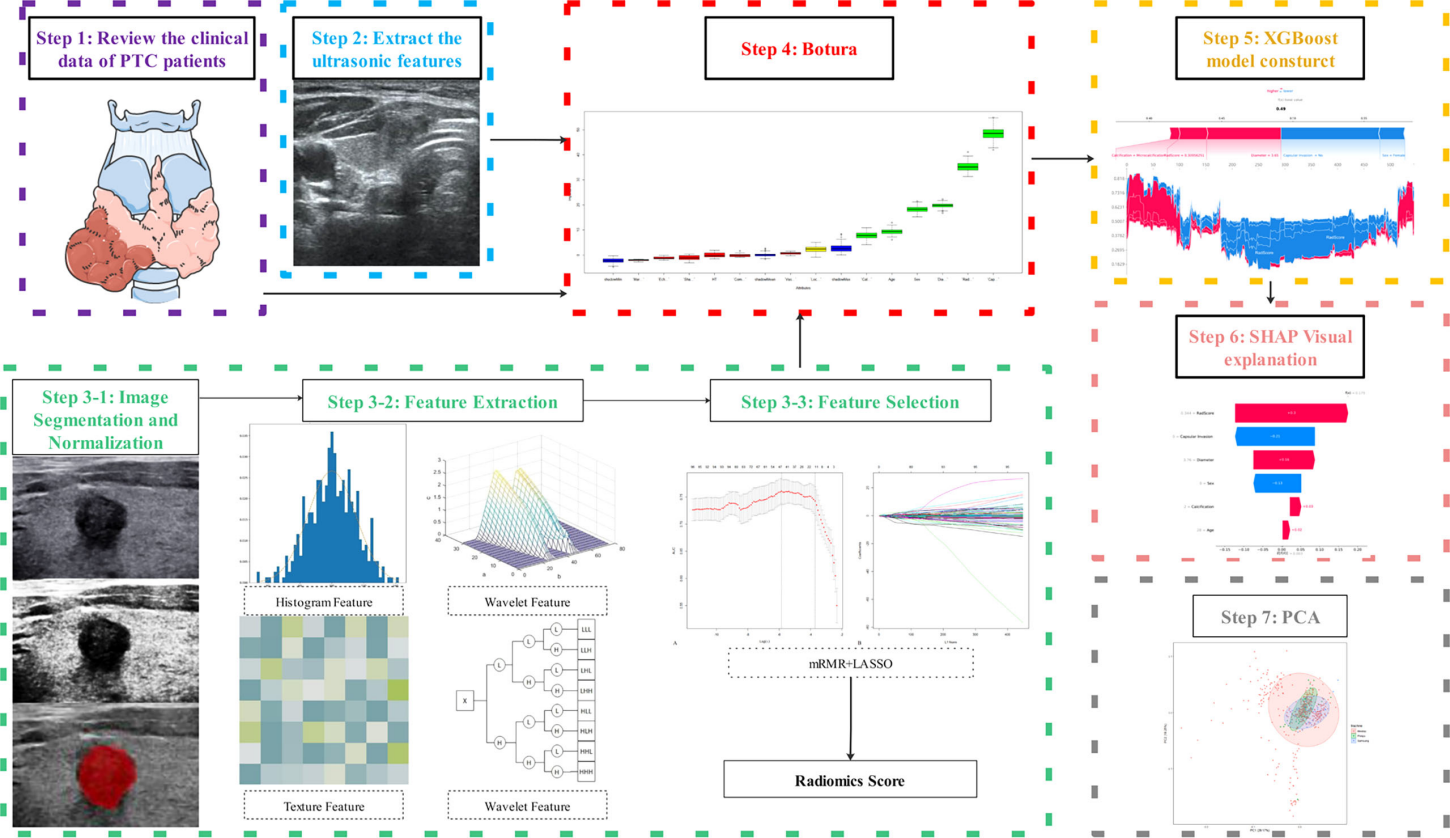
Flowchart of the study.

Original images were exported from the ultrasound imaging workstation in digital imaging and communications in medicine (DICOM) format and imported into ITK-SNAP (version 3.8.0). The polygon tool was used to sketch along the nodule edge to generate regions of interest. The original and segmented images were saved in NRRD format. Histogram equalization was used to preprocess the segmented images (**Appendix 4** and **Appendix Figure 2**). Shape, first-order, texture, wavelet, square root, logarithm, gradient, square, and exponential features were automatically extracted using open-source software (Pyradiomics; http://pyradiomics.readthedocs.io/en/latest/index.html). To ensure the reliability and accuracy of the results, zero-mean normalization (z-score) was performed for data normalization to eliminate index dimension differences of data (19). Features with missing values were deleted; the moderated t-test method was used for difference analysis of the remaining elements. Interclass correlation coefficient (ICC) was used to assess the interobserver agreement of the feature extraction; ICC greater than 0.75 was excellent. A minimum-redundancy maximum relevance (mRMR) was used to remove redundant features. The least absolute shrinkage and selection operator (LASSO) logistic regression method using 10-fold cross-validation was applied to select the most useful predictive CCLNM status-related features from the training cohort. A radiomics score was generated per patient using a linear combination of the chosen features weighted by the LASSO algorithm (**Figure 2**).

### Boruta algorithm: Key features selection

Previous screening methods based on the feature importance of decision trees tended to overestimate the importance of high frequency or high cardinality variables. Therefore, we used the Boruta algorithm, which is suitable for random forest and XGBoost classifiers, to filter out all feature sets used to predict CCLNM status (20). A Boruta algorithm incorporating the independent ultrasound variables, clinical factors, and radiomics score selected the final predictors for CCLNM.

### Establishment and validation of the XGBoost model

Imbalanced data problems significantly degrade the classification performance in machine learning (21). Therefore, we applied the synthetic minority oversampling technique (SMOTE), an oversampling method that randomly generates new instances of minority classes to balance the number of categories (22), during the training process. We used the SMOTE method to generate a perfectly balanced dataset with the same number of instances based on the primary group.

The XGBoost model was constructed based on the final key features screened by the Boruta algorithm. The XGBoost algorithm is described in **Appendix 5**. After the XGBoost model training, a 10-fold cross-validation grid-search method was used to fine-tune the XGBoost algorithm. The balanced accuracy (BA), *F*-score, Matthew’s correlation coefficient (MCC), precision, recall, and area under the receiver operating characteristic (ROC) curve (AUC) were applied to assess the XGBoost model. The model accuracy was evaluated and is presented as root mean square error and coefficient of determination (R^2^). Unsupervised cluster analysis (K-means algorithm) was performed for risk stratification. Decision curve analysis was conducted to estimate net benefits of the XGBoost model at different threshold probabilities. We compared the CCLNM status predicted by the XGBoost model with the status assessed by the radiologist.

### SHAP

SHAP provides a powerful method to measure the importance of features (23, 24) and is introduced to solve the inexplicability bug of machine-learning models. SHAP calculates each variable’s contribution value to the XGBoost model. The SHAP value corresponds to the measure of additive feature attributions. Therefore, the XGBoost model can be visually interpreted globally and locally using SHAP, thus solving the artificial intelligence “black-box” problem.

### Principal components analysis (PCA)

To determine the reliability and reproducibility of the XGBoost model, we assessed potential sources of error during data collection. We assessed the batch effect of ultrasound scanner types on XGBoost using PCA.

### Performance comparison with traditional machine learning models

Six machine learning classifiers, including random forest (RF), artificial neural network (ANN), support vector machine (SVM), decision tree (DT), naive Bayesian (NB), and logistic regression analysis (LRA), were established by the scikit-learn Python library (version 0.24.1). A brief description of these machine learning classifiers is shown in **Appendix 6**. The performance of the XGBoost model was compared with the above six classifiers.

### Statistical analysis

Statistical analyses were performed using R software 4.0.5 and Python Programming Language (version 3.7.9; Python Software Foundation, Wilmington, DE, USA). R software, GraphPad Prism 9.0.0, MedCalc 18.2.1, OriginPro 9.1, and Python were used to create the graphs. A *p*-value less than 0.05 was considered statistically significant. The consistency of PTC ultrasonic image features was evaluated by two radiologists using Kappa. LASSO was performed by R software (glmnet package). Boruta algorithm was operated by R software (Boruta package), and SMOTE was performed by Python imbalanced-learn 0.8.0. The scikit-learn Python library (version 0.24.1) and XGBoost frame (version 1.0.0) were used to establish the XGBoost model in Python. The SHAP Python frame (version 0.39.0) was used to perform SHAP algorithms.

### GitHub

The design code of this study by R software and Python is available on GitHub (https://github.com/shi4180/RadProject).

## Results

### Patient demographics

The baseline epidemiologic and ultrasound image characteristics of the training and test cohorts are listed in **Table 1**. Among the 469 patients in the training cohort, 121 had CCLNM and 348 did not have CCLNM. Of the 118 patients in the test cohort, 32 patients had CCLNM. The Kappa coefficients of the categorical variables were all greater than 0.8 in the consistency analyses; the ICC of diameter was over 0.9 (**Appendix Table 1**).

**Table 1:** Demographics and ultrasound image characteristics in the training and test cohorts.

	Training cohort (N = 469)			Test cohort (N = 118)		
	CCLNM (-)	CCLNM (+)	*p*	CCLNM (-)	CCLNM (+)	*p*
	(n = 348)	(n = 121)		(n = 86)	(n = 32)	
Age	48.4 ± 15.7	43.4 ± 16.0	0.003	51.0 ± 14.0	44.2 ± 13.9	0.021
Sex			0.010			0.127
Female	229 (65.8)	63 (52.1)		58 (67.4)	16 (50.0)	
Male	119 (34.2)	58 (47.9)		28 (32.6)	16 (50.0)	
Diameter (cm)	1.18 ± 0.73	2.06 ± 1.41	<0.001	1.03 ± 0.55	2.30 ± 1.14	<0.001
Location			0.396			0.910
Right lobe	31 (8.91)	16 (13.2)		8 (9.30)	4 (12.5)	
Left lobe	157 (45.1)	52 (43.0)		39 (45.3)	14 (43.8)	
Isthmus	160 (46.0)	53 (43.8)		39 (45.3)	14 (43.8)	
Composition			0.543			1.000
Mixed	56 (16.1)	16 (13.2)		13 (15.1)	4 (12.5)	
Solid	292 (83.9)	105 (86.8)		73 (84.9)	28 (87.5)	
Echogenicity			0.449			0.929
Hyper/Isoechoic	21 (6.03)	4 (3.31)		3 (3.49)	1 (3.12)	
Hypoechoic	188 (54.0)	64 (52.9)		48 (55.8)	17 (53.1)	
Very hypoechoic	139 (39.9)	53 (43.8)		35 (40.7)	14 (43.8)	
Shape			0.744			0.929
Wider-than-tall	136 (39.1)	50 (41.3)		35 (40.7)	14 (43.8)	
Taller-than-wide	212 (60.9)	71 (58.7)		51 (59.3)	18 (56.2)	
Margin			1.000			1.000
Smooth	122 (35.1)	42 (34.7)		34 (39.5)	12 (37.5)	
Ill-defined	212 (60.9)	75 (62.0)		49 (57.0)	19 (59.4)	
Lobulated/irregular	14 (4.02)	4 (3.31)		3 (3.49)	1 (3.12)	
Calcification			<0.001			0.001
None	125 (35.9)	38 (31.4)		24 (27.9)	7 (21.9)	
Macrocalcification	86 (24.7)	60 (49.6)		18 (20.9)	19 (59.4)	
Rim calcification	107 (30.7)	19 (15.7)		33 (38.4)	5 (15.6)	
Microcalcification	30 (8.62)	4 (3.31)		11 (12.8)	1 (3.12)	
Vascularization			0.261			0.666
No	175 (50.3)	53 (43.8)		46 (53.5)	15 (46.9)	
Yes	173 (49.7)	68 (56.2)		40 (46.5)	17 (53.1)	
Capsular invasion			<0.001			<0.001
No	332 (95.4)	57 (47.1)		81 (94.2)	11 (34.4)	
Yes	16 (4.60)	64 (52.9)		5 (5.81)	21 (65.6)	
Radiomics score	0.22 ± 0.09	0.36 ± 0.15	<0.001	0.23 ± 0.09	0.35 ± 0.14	<0.001

CCLNM, central cervical lymph node metastasis.

Data are shown as n (%) or mean ± standard deviation.

### Radiomics feature screening and radiomics score

In the training cohort, 939 features were extracted from the original ultrasound images, including 9 shape features, 18 first-order features, 75 texture features, 372 wavelet features, 93 squareroot features, 93 logarithm features, 93 gradient features, 93 square features, and 93 exponential features. We removed 143 invalid features, and 424 features were screened out from the remaining 796 features after difference analysis (**Appendix Figure 3**). The mRMR algorithm was used to select the 100 most critical features (**Appendix Figure 4**). Next, 11 potential features were chosen among 100 elements in the training cohort with nonzero coefficients in the 10-fold cross-validation LASSO logistic regression model (**Appendix Figure 5** and **Appendix Table 2**). These 11 features were used to calculate the radiomics score. The radiomics scores of CCLNM (+) were 0.36 ± 0.15 and 0.35 ± 0.14 in the training and test cohorts and 0.22 ± 0.09 and 0.23 ± 0.09 for CCLNM (-) patients in the training and test cohorts, respectively (**Table 1** and **Appendix Figure 6**). The performance of CCLNM evaluated by the radiomics score is shown in **Appendix Table 3**.

### Development and performance of the XGBoost model with risk stratification

**Figure 3:**
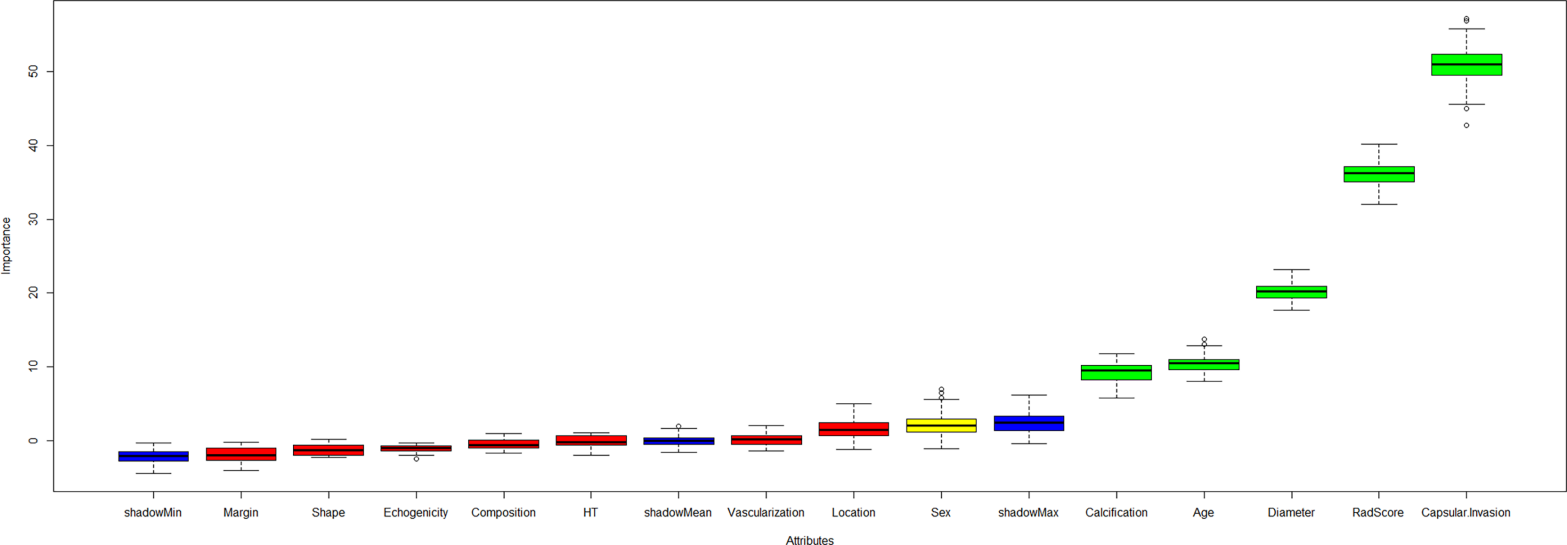
The Boruta algorithm incorporating the independent clinical, ultrasound variables and radiomics score was performed to select the final predictors for CCLNM. The key features included capsular invasion, radiomics score, diameter, age, and calcification.

Five key features were selected by the Boruta algorithm: capsular invasion, radiomics score, diameter, age, and calcification (**Figure 3** and **Appendix Table 4**). The XGBoost model was established based on these five key features.

**Figure 4:**
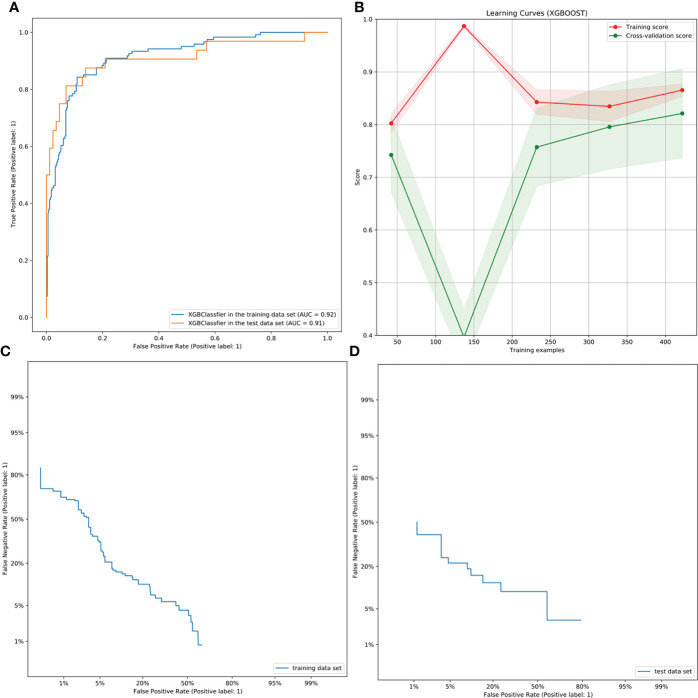
ROC, learning, and DET curves of the XBoost model. **(A)** The ROC curves of the XGBoost model in the training and test cohorts for predicting CCLNM in PTC patients, with an AUC of 0.9153 and 0.9088, respectively. **(B)** The learning curve in the training cohort of the XGBoost model. The two curves finally merge near 0.85, indicating that the model is well fitted for training. **(C, D)** The DET curves of the XGBoost model in the training and test cohorts. They were both concentrated in the third quadrant, indicating that the false rejection rate and false acceptance rate were both low.

The performance of the XGBoost model is shown in **Table 2**. The AUC values of the preoperative assessment of CCLNM in the training and test cohorts were 91.53% and 90.88%, respectively (**Figure 4A**); the BA was over 80% and MCC was more than 60% in the two cohorts. The XGBoost algorithm reflected a good learning curve in the training dataset (**Figure 4B**). The two curves representing the training and test cohorts converged to 0.85, indicating that the XGBoost model effectively prevented overfitting. The detection error tradeoff curves showed that the curves of the two cohorts concentrated in the third quadrant, indicating that the false rejection rate and false acceptance rate were both low (**Figures 4C, D**). The decision curve revealed that if the threshold probability of a physician was over 8%, more advantages would be added by using the XGBoost model to estimate CCLNM in PTC patients (**Appendix Figure 7**).

**Table 2:** Performance of the XGBoost model.

	Training dataset (N = 469)	Test dataset (N = 118)
BA	84.89%	85.21%
*F*-score	73.36%	76.06%
MCC	63.68%	66.56%
Precision	63.10%	69.23%
Recall	87.60%	84.38%
AUC	91.53%	90.88%
RMSE	0.4052	0.3796
R^2^	0.1424	0.2711

XGBoost, eXtreme Gradient Boosting; BA, balanced accuracy; MCC, Matthew’s correlation coefficient; AUC, area under the curve; RMSE, root mean square error; R^2^, coefficient of determination.

Compared with the radiologist, the XGBoost model showed an increased AUC value by 44%. The sensitivity and specificity were also improved to varying degrees (**Appendix Figure 8** and **Appendix Table 5**). The diagnostic sensitivity of the radiologists was only 33.33%, that is to say, many occult metastatic lymph nodes were ignored, and the sensitivity and specificity of the XGBoost model were greatly improved, especially the sensitivity. Therefore, to a certain extent, XGBoost model could spot occult metastatic lymph nodes that cannot be assessed by radiologists.

Along with the XGBoost model, we developed a risk stratification system in the training cohort (**Appendix Figure 9**). All patients were grouped into three categories: low-risk (0-36%), intermediate-risk (37%–58%), and high-risk groups (59%–100%).

### Visual interpretation of the XGBoost model using SHAP

The Sankey plot (**Appendix Figure 10**) showed the orientation of all patients from the predicted CCLNM to the true CCLNM after shunting by age, diameter, calcification, radiomics score, and capsular invasion. The results showed that 19 patients with a predicted negative CCLNM actually had CCLNM. In contrast, 40 patients predicted to have CCLNM were confirmed to be CCLNM (-) based on postoperative pathology (**Appendix Figure 11**).

**Figure 5:**
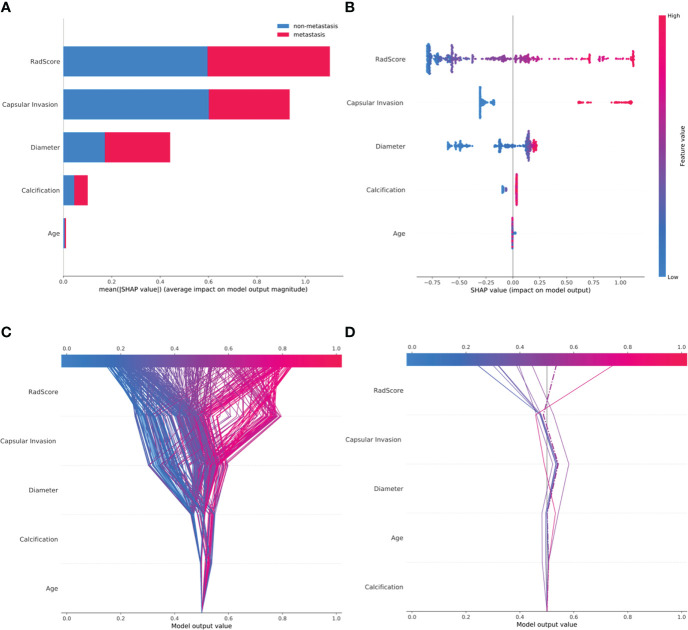
SHAP plots of the XGBoost model. **(A)** The classified bar charts of the SHAP summary plots show the influence of each parameter on the XGBoost model. **(B)** The SHAP summary plot’s scatter plot shows the relationship between the characteristic value and the predicted probability through colors, including positive and negative predictive effects. **(C)** SHAP decision plot for all patients with PTC; **(D)** SHAP decision plot for 10 random patients with PTC, with one misjudgment case (dotted line).

The classified bar chart of the SHAP summary plots was obtained by extracting the average absolute value of SHAP for each parameter, including capsular invasion, radiomics score, diameter, age, and calcification, to show the global significance (**Figure 5A**). The scatter plot of the SHAP summary plot reflects the relationship between the five key parameters and predicted probability through the color, including the positive and negative effects (**Figure 5B**). Radiomics score > 0.3, presence of capsular invasion, diameter > 1.56 cm, male sex, and presence of microcalcification played a positive role in assessing CCLNM. In contrast, older age had a negative effect; that is, patients ≤ 43 years old were more likely to develop CCLNM than patients 43 years old or older.

**Figure 6:**
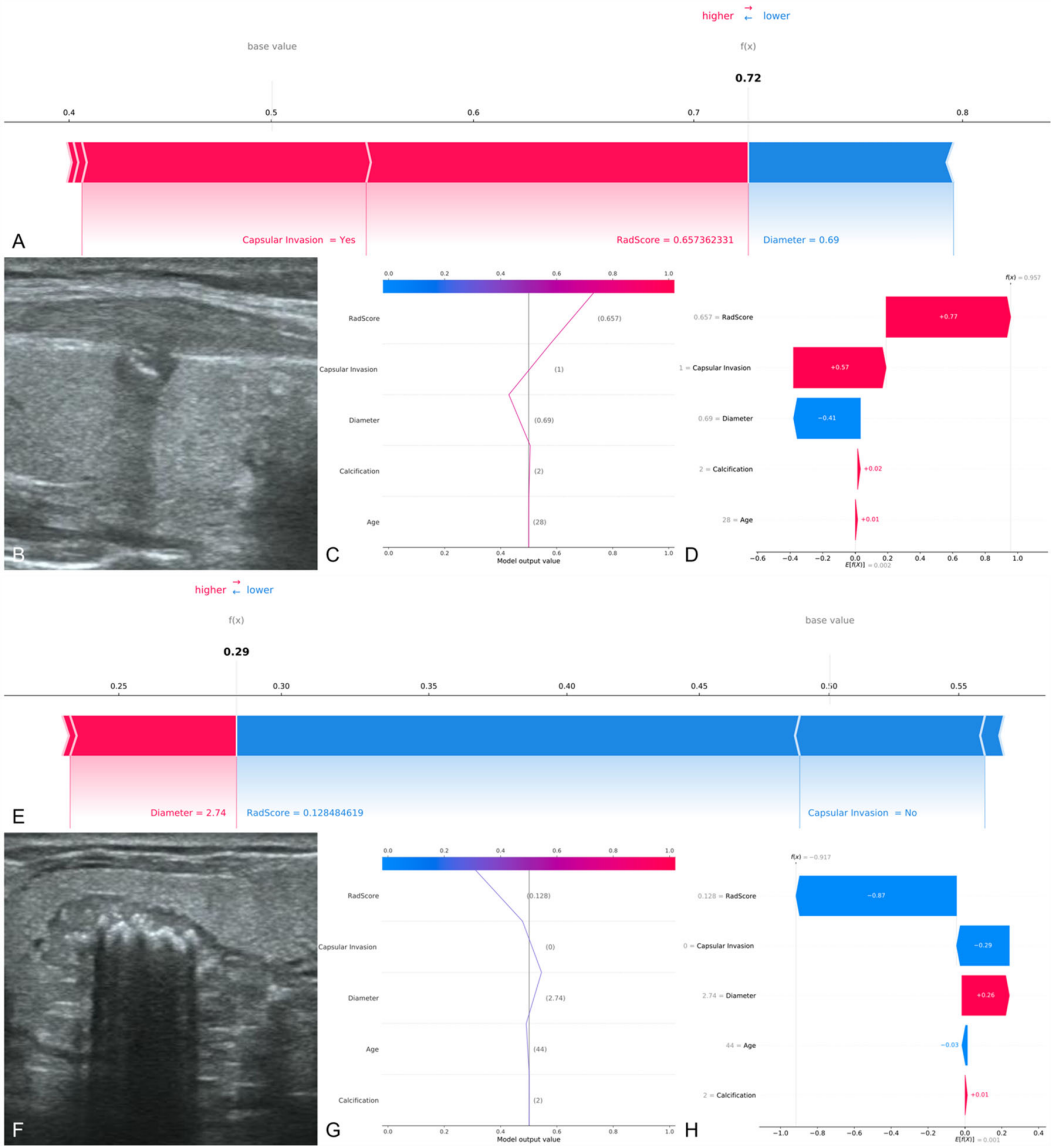
Two examples of correct prediction of CCLNM+ and CCLNM-. **(A–D)** A 28-year-old male patient was admitted to our hospital for further treatment after physical examination found a nodule in the right lobe of the thyroid. Ultrasound examination showed a nodule in the middle of the right lobe of the thyroid, 0.69 cm in diameter, with coarse calcification and capsular invasion. Postoperative pathological findings: papillary carcinoma of the right lobe of thyroid, with lymph node metastasis in the right central region. Retrospective analysis of the case showed that the radiomics score was 0.657 and the XGBoost model predicted CCLNM+ correctly. **(E–H)** A 44-year-old male patient was admitted to our hospital because or volume increase of the right thyroid lobe. Ultrasound examination showed a nodule in the middle of the right lobe of thyroid, 2.74 cm in diameter, with coarse calcification and without capsular invasion. Postoperative pathological findings: papillary carcinoma of the right lobe of thyroid, with no lymph node metastasis in the right central region. Retrospective analysis of the case showed that the 4adiomics score was 0.128 and the XGBoost model predicted CCLNM- correctly.

The SHAP decision plots (**Figures 5C, D**) demonstrated how each critical parameter affected the final decision. Each colored line on the figure represents the predicted outcome for each patient. Moving from the bottom to the top, the SHAP of each element was added to the base value of the XGBoost model, showing how each feature contributed to the overall prediction, including the positive and negative effects. Finally, each line touched the X-axis at the top with its corresponding prediction value, which was the model’s final prediction probability. **Figure 6** shows two examples of the correct prediction of CCLNM(+) and CCLNM(-).

### Evaluation of the XGBoost model stability and repeatability using PCA

To determine the stability and reproducibility of the XGBoost model, we evaluated potential errors during data preparation (25). We assessed the batch effect of ultrasound scanner types on the XGBoost and radiomics score. PCA of the XGBoost for all PTC patients in the training cohort showed no association with the three ultrasound scanner types. This result indicates that the XGBoost model was unaffected by the different types of ultrasound scanners (**Appendix Figure 12**).

### Performance comparison of XGBoost model with six classifiers

In the training cohort, AUCs for RF, ANN, SVM, DT, NB, and LRA were 89.13%, 89.48%, 90.14%, 82.12%, 90.01%, and 88.06%, respectively. Thus, the XGBoost model outperformed other machine learning algorithms in the training cohort (**Appendix Table 6** and **Appendix Figure13**).

## Discussion

In this study, we investigated the feasibility and accuracy of the radiomics-based XGBoost model for prediction of CCLNM in PTC patients based on ultrasound images of the primary tumor. Our study has three significant findings. First, capsular invasion, radiomics score, diameter, age, and calcification were the key features for predicting CCLNM status. Second, the radiomics-based XGBoost model based on the key features showed a favorable ability to discriminate between CCLNM (+) and CCLNM (-), with AUC values of 91.53% and 90.88% in the training and test cohorts, respectively. Third, SHAP provided a reasonable visual interpretation of the prediction, including positive and negative effects.

Ultrasound features, capsular invasion, diameter, and calcification were the critical features for predicting CCLNM. We speculate this may be because of the following reasons. Tumor cells enter the lymph fluid after breaking through the thyroid capsule. When metastatic tumor cells invade the capsule of the lymph nodes, the nodes eventually develop into metastatic lymph nodes (26). A larger tumor diameter indicates a more aggressive tumor. Microcalcification is an indicator of cancer tissue hyperplasia and rapid proliferation of cancer cells; microcalcification may thus be a potential promoter of CCLNM to some extent (27). Previous studies set the age threshold of the PTC patients to 45 or 55 years old according to American Joint Committee on Cancer guidelines (28, 29). In this study, younger age (≤ 43 years) was a key factor for CCLNM, which indicated that PTC occurs in younger patients in recent years.

The radiomics score was independently associated with CCLNM by the Boruta algorithm. Establishing a radiomics score with LASSO has demonstrated excellent results in predicting lymph node metastasis in breast cancer (30), cervical cancer (31), pancreatic carcinoma (32), rectal cancer (33), and lung cancer (34). Radiomics characteristics are closely related to the microstructure and biological behavior of the tumors (31). The radiomics score is based on the high-dimensional and statistical features, which were extracted from primary thyroid tumors. In this study, 11 radiomics features were used to calculate the radiomics score. These features represent the texture information of tumors, which is highly associated with tumor heterogeneity (35). For machine learning, features selection and the reproducibility of the model on different devices are crucial. The Boruta algorithm was used to filter the optimal features, and this algorithm has been recognized by many cutting-edge studies (20, 36). The PCA method was used to ensure the repeatability between different devices, and the result was satisfactory. Therefore, the XGBoost model constructed in this research was also applicable to images obtained by other ultrasound equipment.

Although some studies have used machine learning to predict lymph node metastasis in PTC (6, 37), the “black-box” problem remained to be clarified, that is, an apparent conflict between the performance of the complex model and the clinical interpretability. In this study, the XGBoost model provided a visual interpretation for individual patients using SHAP plots, including positive and negative effects. SHAP considers the impact of a single feature as well as the synergistic effects between features. The XGBoost model not only predicted the possibility of CCLNM but it also provided a rational explanation for the prediction, which may improve clinicians’ confidence in the model.

At present, there are still some CT-based (38–40) and MRI-based (41, 42) radiomics models to predict the condition of cervical lymph nodes in patients with PTC, and they have also achieved good diagnostic performance. However, CT and MRI have certain limitations compared to ultrasound-based radiomics. On the one hand, for CT-based radiomics, tumor diameters < 0.5 cm were not included because they could not be reliably identified and segmented on CT images. The usage of iodinated contrast agents might have the potential to affect the uptake of iodine during the subsequent radioiodine therapy. The increased radiation exposure during contrast-enhanced CT scan should not be ignored. On the other hand, for MRI-based radiomics, the MRI examination involved various elements, including the use of a sequence, magnetic field intensity and some parameters, such as the time of repetition (TR) and the time of echo (TE). The use of MRI radiomics is still a huge challenge due to the complexity of MRI signals, which need to be normalized and standardized.

This study has several limitations. First, this study lacked external validation in other hospitals. The XGBoost model cannot be used for performance verification on other ultrasound equipment. However, we conducted PCA and demonstrated that different equipment did not affect the predictive ability of the model. Second, this study was retrospective in nature. Therefore, we were unable to collect information on the size and number of metastatic lymph nodes. A prospective study is required to confirm the accuracy of the XGBoost model. Third, the peritumoral area was not analyzed, and this should be examined as it might provide information on tumor invasiveness and lymph node metastasis (43). Fourth, the specific location of the lesion, which is close to the upper/lower pole or the anterior/posterior capsule of the thyroid, may have a potential impact on cervical lymph node metastasis. Therefore, the above factors will be included in the next study for in-depth discussion.

In conclusion, we have proposed a radiomics-based XGBoost model for predicting CCLNM in patients with PTC and showed that the model surpassed the evaluation ability of the radiologists. The model integrated ultrasound imaging information with clinical parameters of PTC patients. SHAP provides a reasonable visual interpretation of the XGBoost model to predict CCLNM in patients with PTC. We speculate that the XGBoost model will serve as a promising adjunct in the preoperative evaluation of CCLNM and help assist clinical decision-making for patients with PTC, thereby improving patient prognosis.

## Data availability statement

The raw data supporting the conclusions of this article will be made available by the authors, without undue reservation.

## Author contributions

YS, YZ, JL, and FL contributed to the conception and design of the study and wrote the draft of the manuscript. YW, YC, FS, GC, and XZ organized the database. YZ and XC performed the statistical analysis. All authors contributed to manuscript revision, read, and approved the submitted version.

## Acknowledgments

We thank Medjaden Inc. for scientific editing of this manuscript.

## Conflict of interest

The authors declare that the research was conducted in the absence of any commercial or financial relationships that could be construed as a potential conflict of interest.

## Publisher’s note

All claims expressed in this article are solely those of the authors and do not necessarily represent those of their affiliated organizations, or those of the publisher, the editors and the reviewers. Any product that may be evaluated in this article, or claim that may be made by its manufacturer, is not guaranteed or endorsed by the publisher.
