# Supplementary material for: Ultrasound-based radiomics XGBoost model to assess the risk of central cervical lymph node metastasis in patients with papillary thyroid carcinoma: Individual application of SHAP
Source: Front Oncol. 2022 Aug 26;12:897596. doi: 10.3389/fonc.2022.897596 (PMC9458917; doi:10.3389/fonc.2022.897596)
Supplement: Supplementary file 1 [file Presentation_1.zip › Appendix/Appendix.docx]

**Appendices**

**Appendix 1: The specific principles of operation for patients with papillary thyroid carcinoma (PTC).**

The general principles for the treatment of PTC patients, in department of Thyroid Surgery in Binzhou Medical University Hospital, Shandong, China, were as follows: ① Unilateral lobectomy was performed for primary lesion < 1 cm; ② For primary lesion with a diameter of 1-4cm, total thyroidectomy was considered if preoperative imaging suggested extrathyroid invasion or unilateral lobectomy was considered if the thyroid capsule was intact; ③ For primary lesions > 4 cm, total thyroidectomy should be regarded as; ④ Regardless of the diameter of the primary lesion, total thyroidectomy should be considered if preoperative imaging suggested the presence of lateral cervical lymph node metastasis.

The principles of central cervical lymph node (level Ⅵ) dissection in department of Thyroid Surgery in Binzhou Medical University Hospital were as follows: When the primary lesion was located in the left or right lobe of the thyroid gland, lymph nodes in the ipsilateral central cervical region were dissected. For these patients, we performed ultrasound follow-up for at least one year to confirm that there was no lymph node metastasis in the contralateral central region. When the primary lesion was located in the isthmus or proximal isthmus, bilateral central lymph node dissection was performed.

**Appendix 2: The detailed ultrasound protocols.**

All included ultrasound images were re-evaluated by two radiologists, in the department of Ultrasound in Binzhou Medical University Hospital, Shandong, China, with 11 and 13 years of experience in thyroid cancer ultrasound diagnosis. PHILIPS EPIQ5 equipping with L12-5 linear array probe and 5-12MHz frequency, SAMSUNG RS80A equipping with L3-12A linear array probe and 3-12MHz frequency, and mindray DC8 equipping with L12-3E linear array probe and 7.5-12MHz frequency were used to perform the examination. All patients were scanned in the supine position with shoulder pillows and their necks fully exposed; along with longitudinal and transverse scans, routine examination of the bilateral thyroid lobe, isthmus, and bilateral cervical lymph nodes was performed.

**Appendix 3: Specific ultrasound images analysis method.**

The parameters from ultrasound images were re-evaluated independently by two head and neck radiologists, in the department of Ultrasound in Binzhou Medical University Hospital, Shandong, China, with 11 and 13 years of clinical experience who were blinded to the clinical information and pathological diagnosis.

Diameter and shape (wider-than-tall or taller-than-wide) were measured using Image Pro Plus (IPP) software (Media Cybernetics, Silver Spring, USA). Manual freehand delineation of a line was performed on three adjacent slices containing the largest lesion area to measure the diameter of each PTC lesion (Appendix Figure 1A). Diameter was measured three times by the two radiologists and averaged, respectively. The final measurement result was the average of the average values measured by two radiologists at the first time. A week later, the diameters of all lesions were re-test. Intra- and inter-observer consistency analyses were performed by Kappa.

Except for diameter, other parameters were all categorical variables. Taller-than-wide was defined as the anteroposterior diameter of the nodule that was larger than its transverse diameter on a transverse plane (Appendix Figure 1B) (1). Calcification in the PTC lesion was divided into four forms: none and significant comet-tail calcification, macrocalcification, rim calcification, and microcalcification. According to Thyroid Imaging Reporting and Data System (ACR TI-RADS) (2), microcalcification appears very small (≤1mm) hyperechoic regions (1), macrocalcification appears was more extensive (>1mm) hyperechoic regions, and rim calcification was annular calcification in the tumor margin that looks like an eggshell (Appendix Figure 1C-E) (3). The capsular invasion was defined as the disruption of the perithyroidal echogenic line between the primary site of PTC and the standard thyroid capsule on ultrasound (Appendix Figure 1F) (4). The two radiologists evaluated the above parameters, respectively. When there was a disagreement, they reported to the superior doctor, a chief physician with more than 20 years of work experience, and reached an agreement after discussion.

**Appendix 4: Histogram equalization**

Different ultrasound equipment and ultrasonic scanning parameters, such as scan frequency, gain, time gain compensation, dynamic range, or scan depth, will lead to different gray values and distribution of the collected ultrasonic images, resulting in different image omics features. On the other hand, ultrasonic images are characterized by more speckle noise, blurred edges, low contrast and uneven gray scale. All of the above reasons could lead to the reduction of the generalization performance of the constructed image omics model, thus reducing the stability and repeatability of the model. In order to solve the above problems, this study, in Binzhou Medical University Hospital, Shandong, China, uses histogram equalization to preprocess the extracted images.

Histogram equalization method is a simple and effective image enhancement technology (5), which is mainly used to enhance the contrast of images with small dynamic range by changing the histogram of the image to change the gray level of each pixel in the image. The original image is not clear because its gray distribution may be concentrated in a narrow range. For example, the gray level of an overexposed image is concentrated in the high brightness range, while underexposure will concentrate the gray level of the image in the low brightness range. Histogram equalization can transform the histogram of the original image into the form of uniform distribution, so as to increase the dynamic range of gray value difference between pixels, so as to enhance the overall contrast of the image. Histogram equalization, in short, the basic principle, namely: the number of pixels in the image of gray value (that is, a major role on the image gray value) for broadening, and a small number of pixels of gray value (that is, a minor role on the picture of the gray value) to merge, thus increasing the contrast, make the image clear, reach the purpose of enhancing (6).

**Appendix 5: A detailed introduction to the construction of the eXtreme Gradient Boosting (XGBoost) algorithm.**

When dealing with small and medium structured data, the decision tree algorithm is the best. Additionally, XGBoost is an integrated machine-learning algorithm based on decision trees, framed by Gradient Boost. To train the model, we should define the objective function to measure the fitting degree of the model to the training data. The salient characteristic of objective functions is that it consists of training loss and a regularization term. Moreover, the XGBoost technique optimizes both parts of the model. In the study, we used XGBoost to predict the possibility of central cervical lymph node metastasis (CCLNM). “$y_{i}$” for the given input feature vectors $X_{i}$= ($X_{1}$, $X_{2}$, ……, $X_{N}$) including preoperative ultrasound and DECT parameters. The training process is conducted through additional strategies. Given a residue $i$ with $X_{i}$, a tree ensemble model uses $K$ additive functions to predict the output value $\hat{y}_{i}$ as follows:

$\hat{y}_{i}= \sum_{k=1}^{K} f_{k} (X_{i}),f_{k} \in F$ (1)

where $f_{k} (X_{i})$ denotes an independent tree structure with leaf scores of $X_{i}$, and $F$ denotes the space of trees. The XGBoost algorithm introduces the regular function to control overfitting. The objective part of XGBoost is defined as follows:

$obj=\sum_{i} l(y_{i}, \hat{y}_{i})+\sum_{t} \Omega(f_{t})$ (2)

where $l(y_{i}, \hat{y}_{i})$ denotes the training loss function, and $\Omega$ denotes the regularization term:

$\Omega\left( f_{t} \right)= \gamma T+\frac{1}{2}\sum_{j=1}^{T} \omega_{j}^{2}$ (3)

where $T$ denotes the number of leaves, and $\omega_{j}$ denotes the leaf node output in each sub decision tree model. The variables $\gamma$and are constants that control the degree of regularization.

Then, the XGBoost takes the Taylor expansion of the loss function up to the second order and removes all constants, so the specific objective at step $t$ becomes:

${obj}^{(t)}=\sum_{i=1}^{n} \left[ g_{i}f_{t}\left( x_{i} \right)+\frac{1}{2}h_{i}f_{t}^{2}\left( x_{i} \right) \right]+ \Omega(f_{t})$ (4)

where $g_{i}$ and $h_{i}$are defined as follows:

$g_{i}=\partial_{\hat{y}_{i}^{(t-1)}}l(y_{i},\hat{y}_{i}^{(t-1)})$ (5)

$h_{i}=\partial_{\hat{y}_{i}^{(t-1)}}^{2}l(y_{i},\hat{y}_{i}^{(t-1)})$ (6)

The value of the objective function depends only on $g_{i}$ and $h_{i}$ and can optimize every loss function, including logistic regression and pairwise ranking.

An essential advantage of the definition is that the value of the objective function depends only on $g_{i}$ and $h_{i}$.This is how XGBoost supports custom loss functions. We can use the same solver to optimize each loss function by inputting $g_{i}$ and $h_{i}$, including logistic regression and pairwise ranking. As a result, the XGBoost model provides a more accurate prediction model and effectively prevents overfitting.

**Appendix 6: A brief description of six machine learning classifiers**

Random forest is an ensemble machine learning method for classification and regression, which operated by constructing a large number of decision trees and outputting classes as a single tree (classification) or average prediction (regression) model.

Artificial neural network is a computational model based on the structure and function of biological neural networks. The main advantage of ANN was the ability to approximate any nonlinear mathematical function.

Support vector machine is a machine learning approach which is based on the structural risk minimization principle of statistics learning. It projected data into a multidimensional space and classified it with hyper-planes.

Decision tree is a basic classification and regression method. C5.0 was one of the classic decision tree model algorithms, which can generate multi-branch decision trees, and the target variables were categorical.

Naive Bayesian is a corresponding simplification on the basis of Bayesian algorithm, that is, when it is assumed that the target value is given, the attributes are conditionally independent of each other. In practical application scenarios, the complexity of Bayesian methods is greatly simplified.

Logistic regression analysis is a statistical method which is used to analyze a data set in which one or more independent variables that determine the outcome. The outcome was measured by a dichotomous variable, in which there were only two possible outcomes.

**Appendix Table 1: Inter-observers consistency analysis in the training cohort**

|  | Kappa coefficient/ICC | 95% CI |
| --- | --- | --- |
| Diameter^*^ | 0.9989 | 0.9987 - 0.9991 |
| Location | 1.00000 | 1.00000-1.00000 |
| Composition | 1.00000 | 1.00000-1.00000 |
| Echogenicity | 0.89209 | 0.85750-0.92667 |
| Shape | 0.89703 | 0.86050-0.93356 |
| Margin | 0.86912 | 0.82901-0.90923 |
| Calcification | 0.95778 | 0.94329-0.97228 |
| Vascularization | 1.00000 | 1.00000-1.00000 |
| Capsular invasion | 0.88043 | 0.83037-0.93050 |

ICC, Intraclass correlation coefficient; CI, confidence interval.

^*^: ICC was used in the consistency analysis of continuous variable.

**Appendix Table 2: Features screened out by 10-fold cross-validation LASSO regression**

| Radiomics features | LASSO coefficient |
| --- | --- |
| logarithm_glcm_Idm | -0.118511230 |
| wavelet-LL_glrlm_LowGrayLevelRunEmphasis | -0.107334396 |
| original_firstorder_Skewness | 0.217332227 |
| wavelet-HL_glszm_GrayLevelNonUniformity | 0.411190309 |
| wavelet-LH_firstorder_Variance | 0.083898623 |
| exponential_gldm_SmallDependenceEmphasis | -0.118682556 |
| wavelet-LH_glszm_GrayLevelNonUniformityNormalized | 0.064629599 |
| wavelet-HL_glszm_GrayLevelNonUniformityNormalized | 0.115087157 |
| wavelet-LH_glcm_Imc2 | -0.581750304 |
| wavelet-HH_glcm_ClusterProminence | -0.165206744 |
| wavelet-HL_glszm_SizeZoneNonUniformity | 0.009068561 |

LASSO, least absolute shrinkage and selection operator.

**Appendix Table 3: The performance of radiomics score**

|  | Training cohort | Test cohort |
| --- | --- | --- |
| Binomial Deviance | 1.48094 | 1.475688 |
| Misclassification Error | 0.7420043 | 0.7288136 |
| AUC (95% CI) | 0.7792 (0.7301-0.8283) | 0.7624 (0.6556-0.8691) |
| Mean Squared Error | 0.5473113 | 0.5447197 |
| Mean Absolute Error | 1.037338 | 1.034789 |

AUC, area under the curve; CI, confidence interval.

**Appendix Table 4: Key features selection using Boruta algorithm**

|  | meanImp | medianImp | minImp | maxImp | normHits | decision |
| --- | --- | --- | --- | --- | --- | --- |
| Age | 10.32431 | 10.45232 | 7.995779 | 13.74561 | 1 | Confirmed^*^ |
| Sex | 2.138669 | 2.055599 | -1.12528 | 6.95742 | 0.414141 | Tentative |
| Diameter | 20.18624 | 20.20805 | 17.6279 | 23.16552 | 1 | Confirmed^*^ |
| Location | 1.575864 | 1.451098 | -1.2516 | 5.013222 | 0.30303 | Rejected |
| Composition | -0.48906 | -0.64908 | -1.72418 | 0.912812 | 0 | Rejected |
| Echogenicity | -1.15411 | -1.01241 | -2.50697 | -0.3509 | 0 | Rejected |
| Shape | -1.2650621 | -1.3175289 | -2.336663 | 0.1410719 | 0 | Rejected |
| Margin | -1.99419 | -1.97633 | -4.08668 | -0.19816 | 0 | Rejected |
| Calcification | 9.204321 | 9.532025 | 5.74205 | 11.77026 | 1 | Confirmed^*^ |
| Vascularization | 0.182087 | 0.204858 | -1.44132 | 2.049119 | 0 | Rejected |
| Capsular invasion | 50.83545 | 50.9989 | 42.77423 | 57.17354 | 1 | Confirmed^*^ |
| Radiomics score | 36.22215 | 36.25635 | 32.02534 | 40.17172 | 1 | Confirmed^*^ |

^*^: Six key features selection using Boruta algorithm.

**Appendix Table 5: Comparison of the performance between the XGBoost model and radiologist in predicting the CCLNM status.**

|  | XGBoost | Radiologist | *P* value |
| --- | --- | --- | --- |
| AUC | 0.91 (0.843 - 0.955) | 0.532 (0.438 - 0.625) | <0.0001 |
| Sensitivity | 75.76% | 33.33% | 0.0001 |
| Specificity | 91.76% | 74.12% | 0.0081 |
| Positive predictive value | 78.13% | 33.33% | - |
| Negative predictive value | 90.70% | 74.12% | - |
| Accuracy | 87.29% | 62.71% | - |

Note: 95% confidence interval in parentheses.

XGBoost, explainable eXtreme Gradient Boosting; CCLNM, central cervical lymph node metastasis; AUC, area under the curve.

**Appendix Table 6: Prediction performance of the six machine learning classifiers**

|  | BA | *F*-score | MCC | precision | recall | R^2^ | RMSE | AUC |
| --- | --- | --- | --- | --- | --- | --- | --- | --- |
| RF | 82.09% | 71.76% | 61.24% | 66.67% | 77.69% | 0.18 | 0.40 | 89.13% |
| ANN | 84.82% | 77.37% | 69.45% | 77.05% | 77.69% | 0.39 | 0.34 | 89.48% |
| SVM | 82.90% | 72.39% | 62.06% | 65.99% | 80.17% | 0.18 | 0.40 | 90.14% |
| DT | 83.44% | 70.63% | 60.05% | 58.79% | 88.43% | 0.0087 | 0.44 | 82.12% |
| NB | 81.75% | 72.65% | 63.00% | 71.77% | 73.55% | 0.25 | 0.38 | 90.01% |
| LRA | 82.09% | 69.62% | 58.27% | 59.30% | 84.30% | 0.0087 | 0.44 | 88.06% |

RF, random forest; ANN, artificial neural network; SVM, support vector machine; DT, decision tree; NB, naive Bayesian; LRA, logistic regression analysis; BA, balanced accuracy; MCC, Matthew’s correlation coefficient; R^2^, coefficient of determination; RMSE, root mean square error; AUC, area under the curve.

**Appendix Figure 1: Specific ultrasound images analysis method.**

A, The diameter was defined as the longest diameter of the nodule. B, Taller-than-wide was defined as the anteroposterior diameter of the nodule that was larger than its transverse diameter on a transverse plane. C, Microcalcification in the nodule. D, Macrocalcification in the nodule. E, Rim calcification in the nodule. F, The capsular invasion was defined as the disruption of the perithyroidal echogenic line between the primary site of PTC and the standard thyroid capsule on ultrasound. PTC, papillary thyroid carcinoma.

**Appendix Figure 2: Diagram of histogram equalization.**

**Appendix Figure 3: Difference analysis for 424 features.**

**Appendix Figure 4: The heatmap of the most important 424 features.**

**Appendix Figure 5: Feature selection using the LASSO logistic regression model**

A, Tuning parameter (λ) selection in the LASSO model used 10-fold cross-validation for 469 PTC patients. The mean deviance (red dots) was plotted versus log(λ), error bars displaying the range of standard error. B, LASSO coefficient profiles of the 100 features. LASSO coefficient profiles plotted versus log(λ), gray vertical line was drawn at the value selected using 10-fold cross validation, where the optimal λ resulted in 11 nonzero coefficients. LASSO, least absolute shrinkage and selection operator; PTC, papillary thyroid carcinoma

**Appendix Figure 6: Radiomics score distribution in the training (A) and test (B) cohorts, the green represented CCLNM (-), the red represented CCLNM (+).**

CCLNM, central cervical lymph node metastasis.

**Appendix Figure 7: Decision curve analysis of the XGBoost model. XGBoost, eXtreme Gradient Boosting.**

**Appendix Figure 8: The ROC curves of CCLNM predicted by XGBoost model and radiologist.** ROC, receiver operating characteristic; CCLNM, central cervical lymph node metastasis; XGBoost, explainable eXtreme Gradient Boosting.

**Appendix Figure 9: Risk stratification system of the XGBoost model in the training cohort.**

All patients were grouped into three categories: low-risk (0-36%), intermediate-risk (37%-58%), and high-risk (59%-100%). XGBoost, explainable eXtreme Gradient Boosting.

**Appendix Figure 10: Sankey plot showed the orientation of all patients from the predicted CCLNM to the true CCLNM.**

Among the continuous variables, including age, diameter, and Radiomics score, the cut-off value was calculated according to the sensitivity and specificity of the ROC curve. CCLNM, central cervical lymph node metastasis. ROC, receiver operating characteristic.

**Appendix Figure 11: Confusion matrix of the training cohort.**

**Appendix Figure 12: A PCA plot of XGBoost model by different ultrasound scanner manufacturers.**

It showed the key features of the primary tumors had no association to the three ultrasound devices. PCA, principal component analysis; XGBoost, explainable eXtreme Gradient Boosting

**Appendix Figure 13: The mixed ROC curves of the six machine learning models.**

ROC, receiver operating characteristic; RF, random forest; ANN, artificial neural network; SVM, support vector machine; DT, decision tree; NB, naive Bayesian; LRA, logistic regression analysis.

**References:**

1. Won-Jin Moon, So Lyung Jung, Jeong Hyun Lee, Dong Gyu Na, Jung-Hwan Baek, Young Hen Lee, et al. Benign and Malignant Thyroid Nodules: US Differentiation-Multicenter Retrospective Study. Radiology. 2008;247(3):762-70. doi: 10.1148/radiol.2473070944.

2. Tessler FN, Middleton WD, Grant EG, Hoang JK, Berland LL, Teefey SA, et al. ACR Thyroid Imaging, Reporting and Data System (TI-RADS): White Paper of the ACR TI-RADS Committee. J Am Coll Radiol. 2017;14(5):587-95. Epub 2017/04/05. doi: 10.1016/j.jacr.2017.01.046.

3. Gharib H, Papini E, Garber JR, Duick DS, Harrell RM, Hegedus L, et al. American Association of Clinical Endocrinologists, American College of Endocrinology, and Associazione Medici Endocrinologi Medical Guidelines for Clinical Practice for the Diagnosis and Management of Thyroid Nodules--2016 Update. Endocr Pract. 2016;22(5):622-39. Epub 2016/05/12. doi: 10.4158/EP161208.GL.

4. Wei X, Wang M, Wang X, Zheng X, Li Y, Pan Y, et al. Prediction of cervical lymph node metastases in papillary thyroid microcarcinoma by sonographic features of the primary site. Cancer biology & medicine. 2019;16(3):587-94. Epub 2019/10/01. doi: 10.20892/j.issn.2095-3941.2018.0310.

5. Subramani B, Veluchamy M. Fuzzy Gray Level Difference Histogram Equalization for Medical Image Enhancement. J Med Syst. 2020;44(6):103. Epub 2020/04/21. doi: 10.1007/s10916-020-01568-9.

6. Singh P, Mukundan R, De Ryke R. Feature Enhancement in Medical Ultrasound Videos Using Contrast-Limited Adaptive Histogram Equalization. Journal of digital imaging. 2020;33(1):273-85. Epub 2019/07/05. doi: 10.1007/s10278-019-00211-5.
