# Supplementary figures and images for: Ultrasound-based radiomics XGBoost model to assess the risk of central cervical lymph node metastasis in patients with papillary thyroid carcinoma: Individual application of SHAP
Source: Front Oncol. 2022 Aug 26;12:897596. doi: 10.3389/fonc.2022.897596 (PMC9458917; doi:10.3389/fonc.2022.897596)

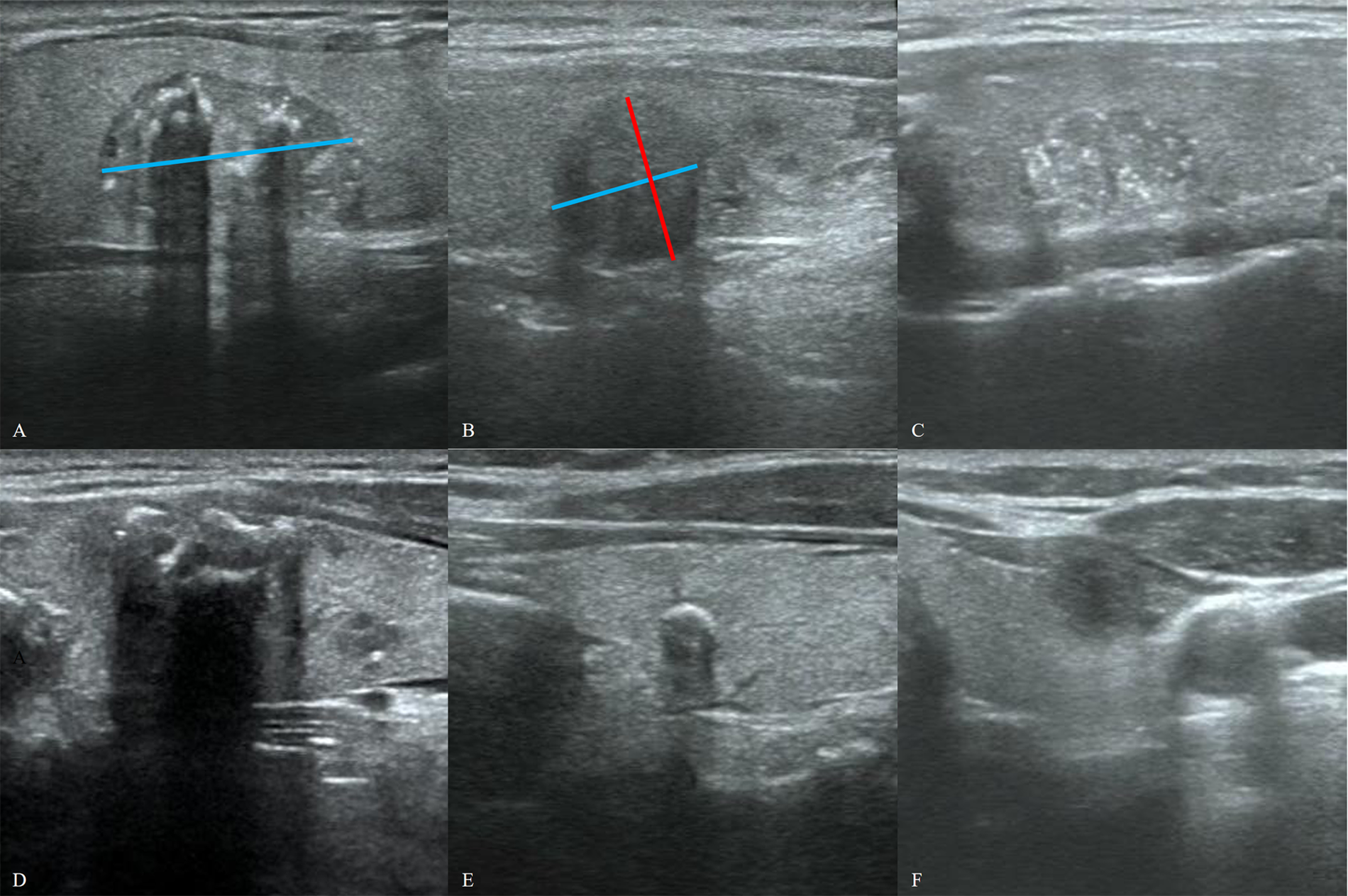

Supplement: Supplementary file 1 [file Presentation_1.zip › Appendix/Appendix Figures/Appendix Fig. 1.tif]

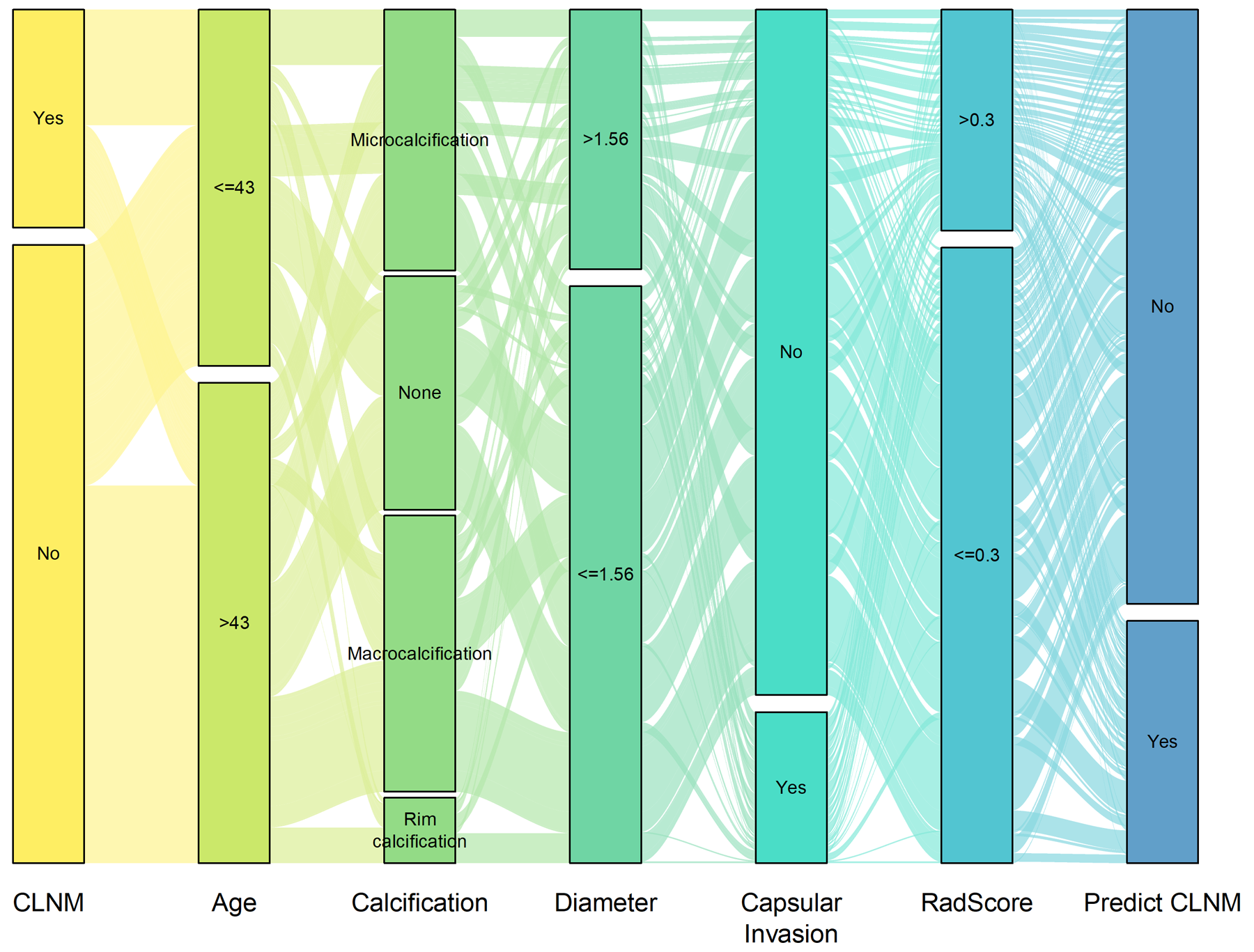

Supplement: Supplementary file 1 [file Presentation_1.zip › Appendix/Appendix Figures/Appendix Fig. 10 sankeyplot.tif]

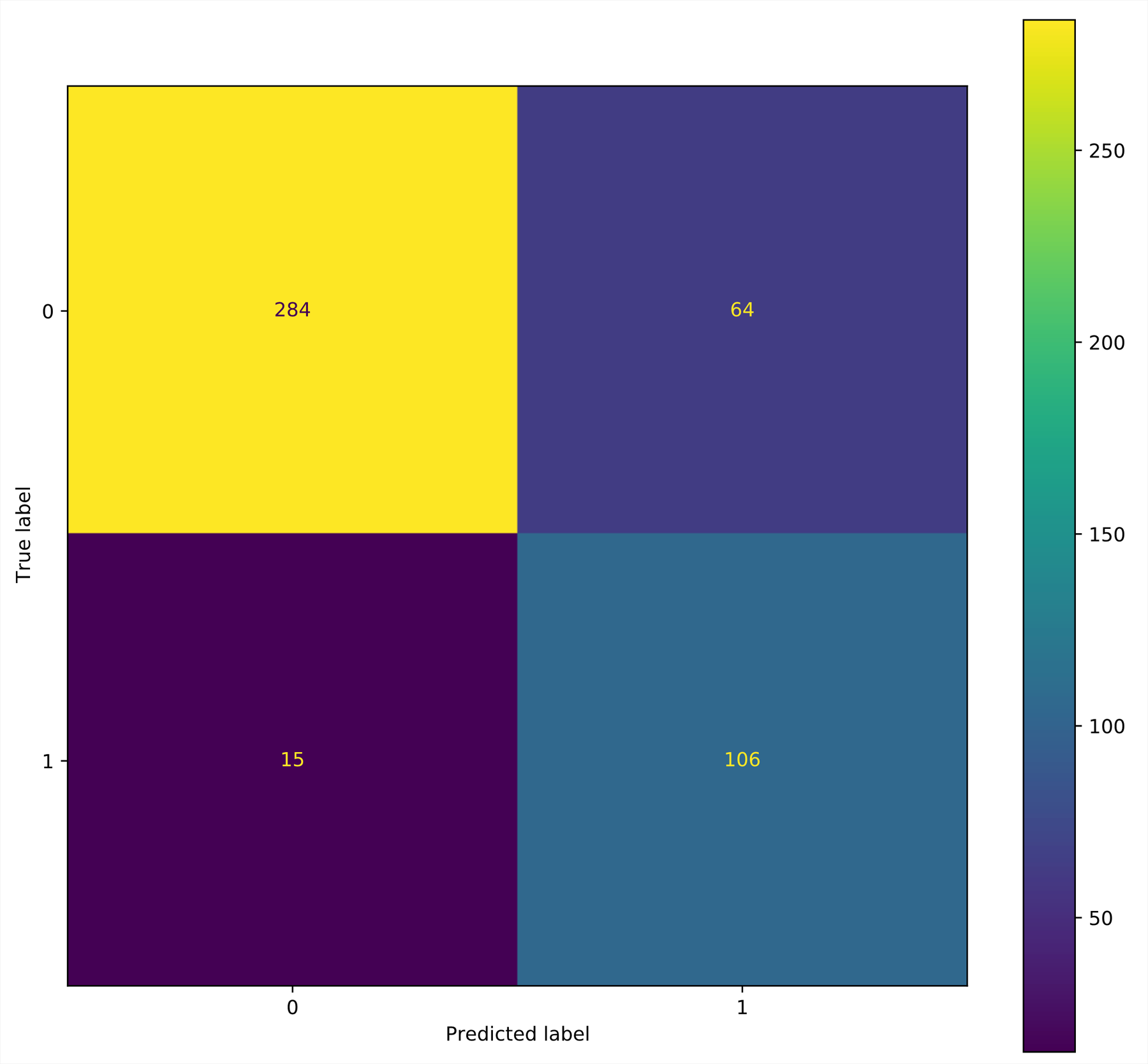

Supplement: Supplementary file 1 [file Presentation_1.zip › Appendix/Appendix Figures/Appendix Fig. 11 ConfusionMatrixTrain.tif]

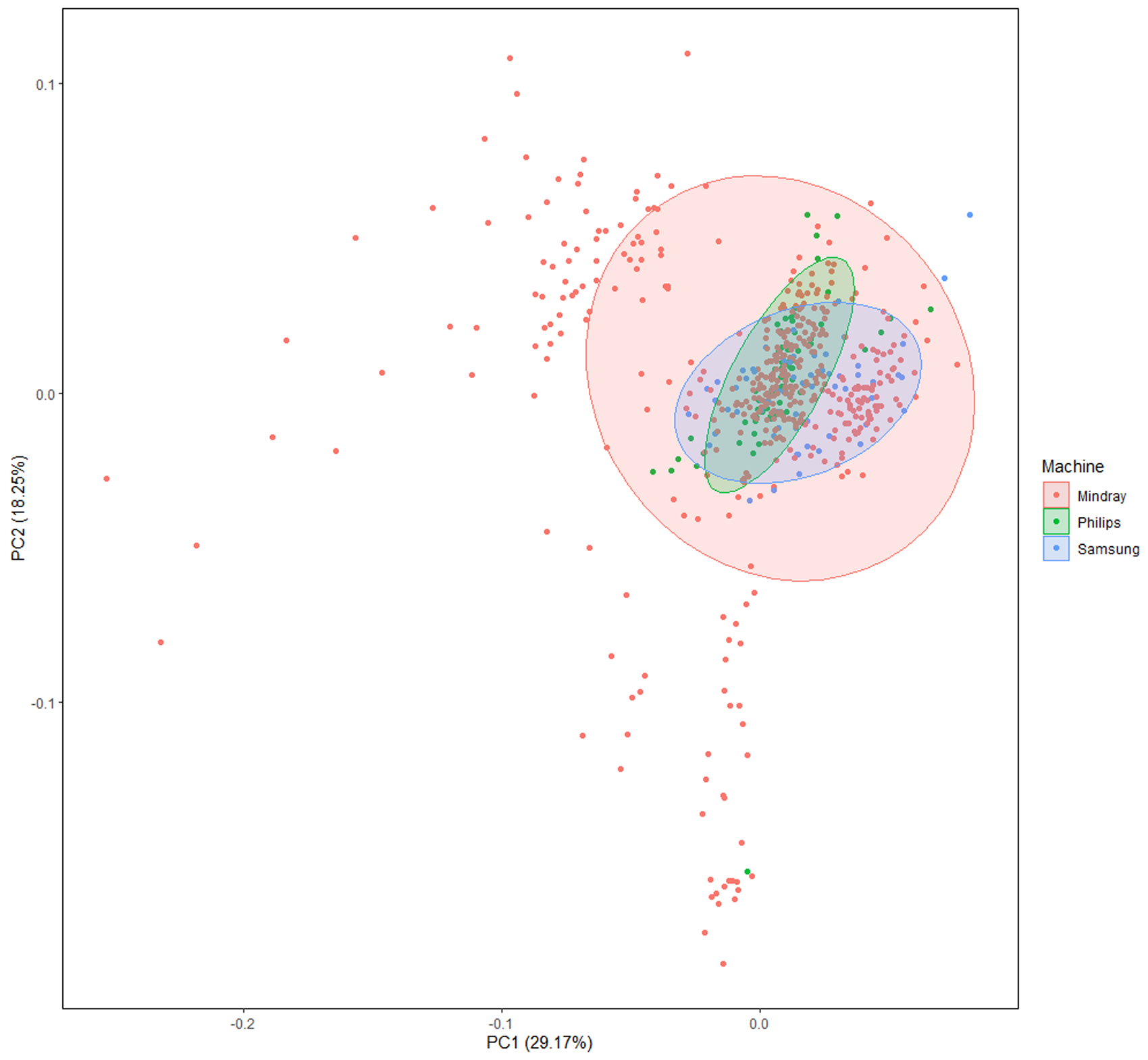

Supplement: Supplementary file 1 [file Presentation_1.zip › Appendix/Appendix Figures/Appendix Fig. 12 PCAPlot.tiff]

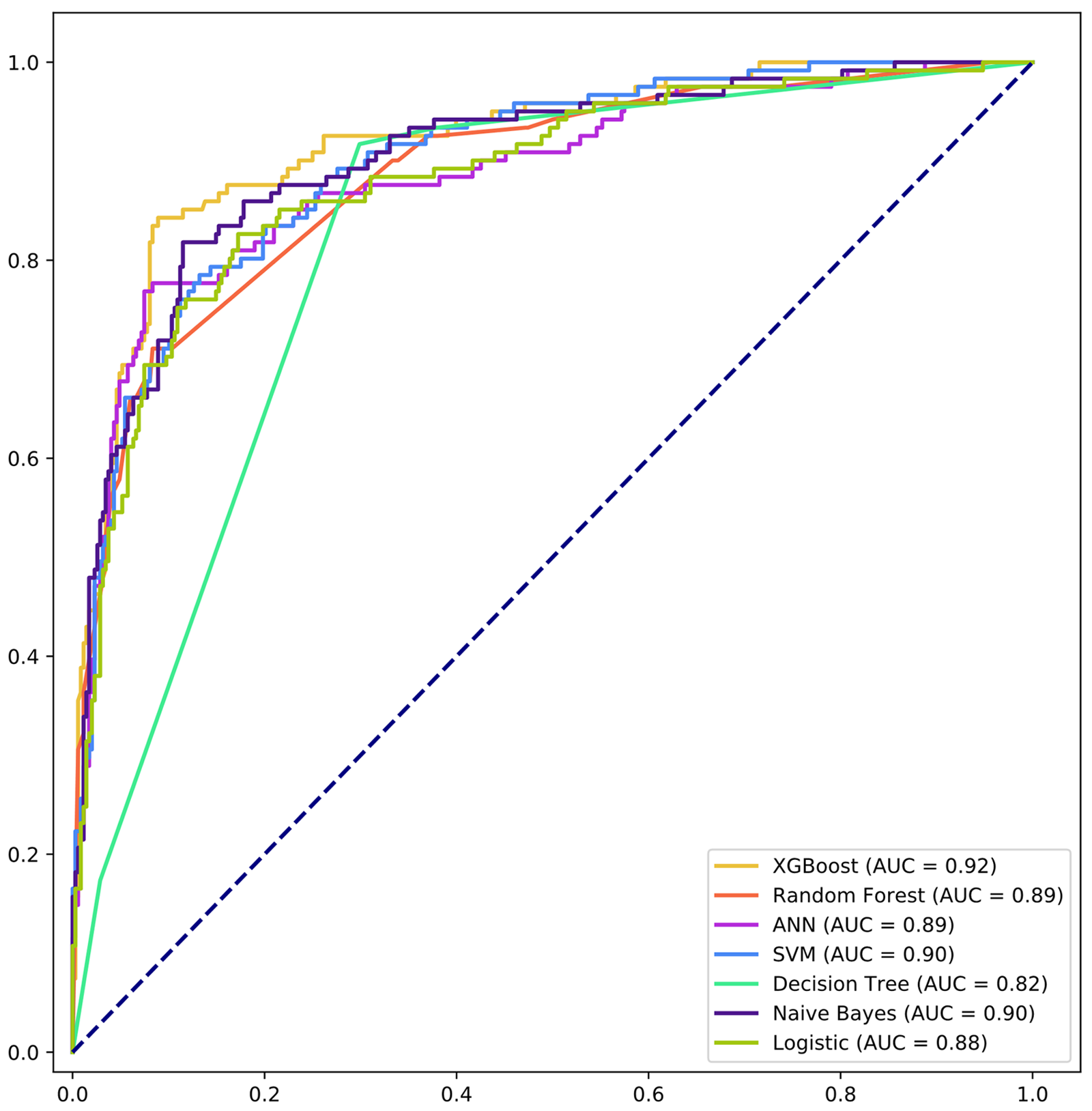

Supplement: Supplementary file 1 [file Presentation_1.zip › Appendix/Appendix Figures/Appendix Fig. 13.TIF]

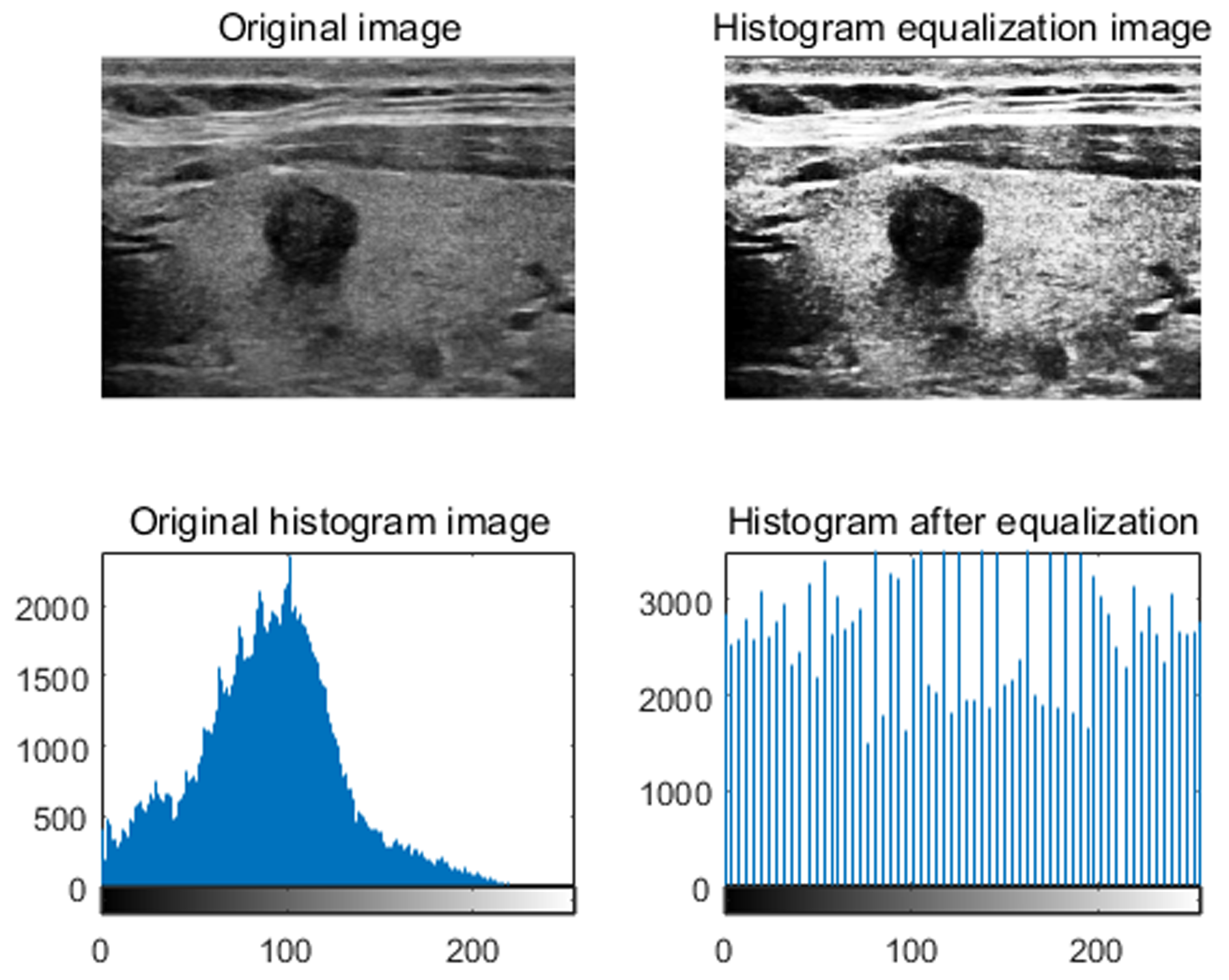

Supplement: Supplementary file 1 [file Presentation_1.zip › Appendix/Appendix Figures/Appendix Fig. 2 Histogram Plot.tif]

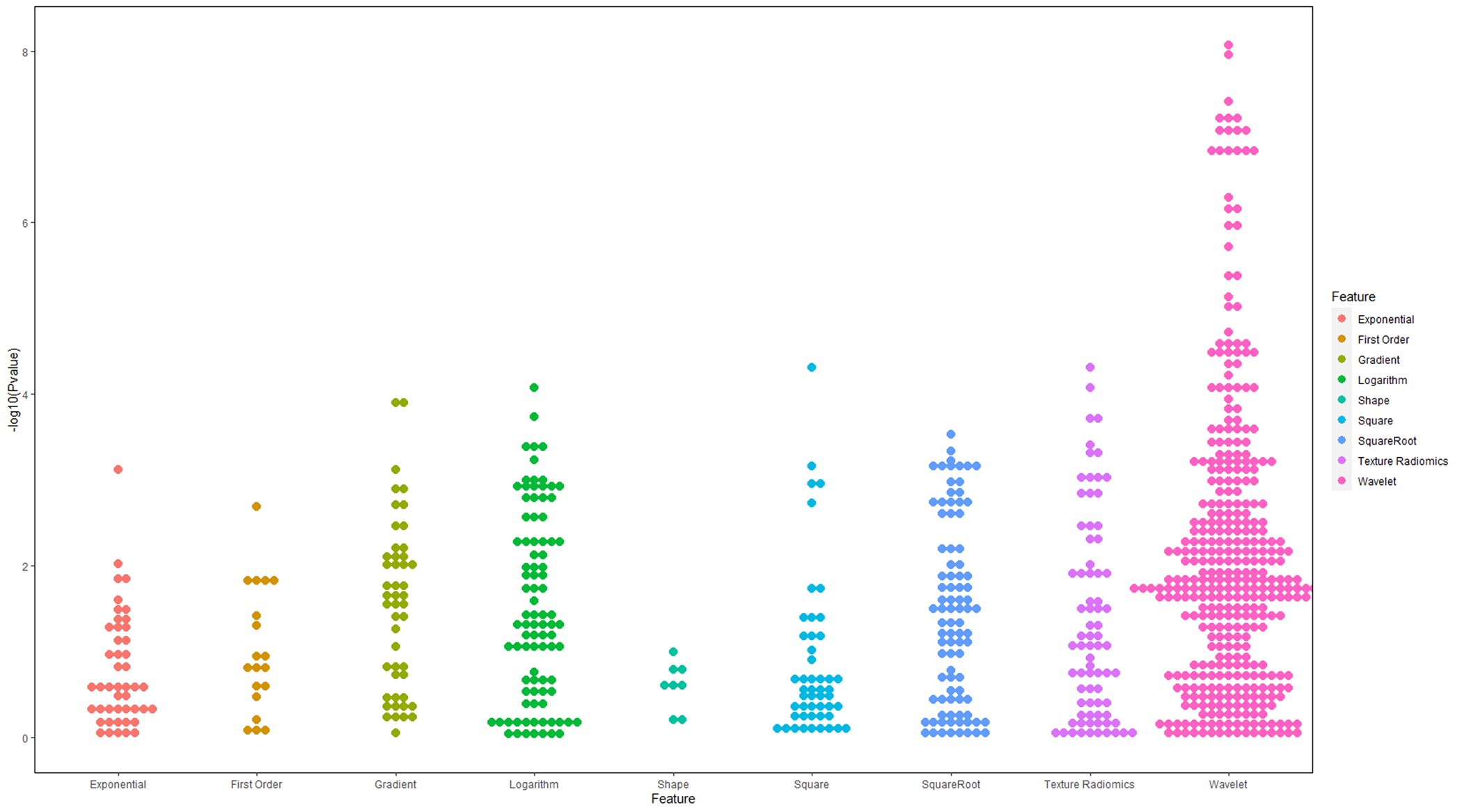

Supplement: Supplementary file 1 [file Presentation_1.zip › Appendix/Appendix Figures/Appendix Fig. 3 dotplot.tiff]

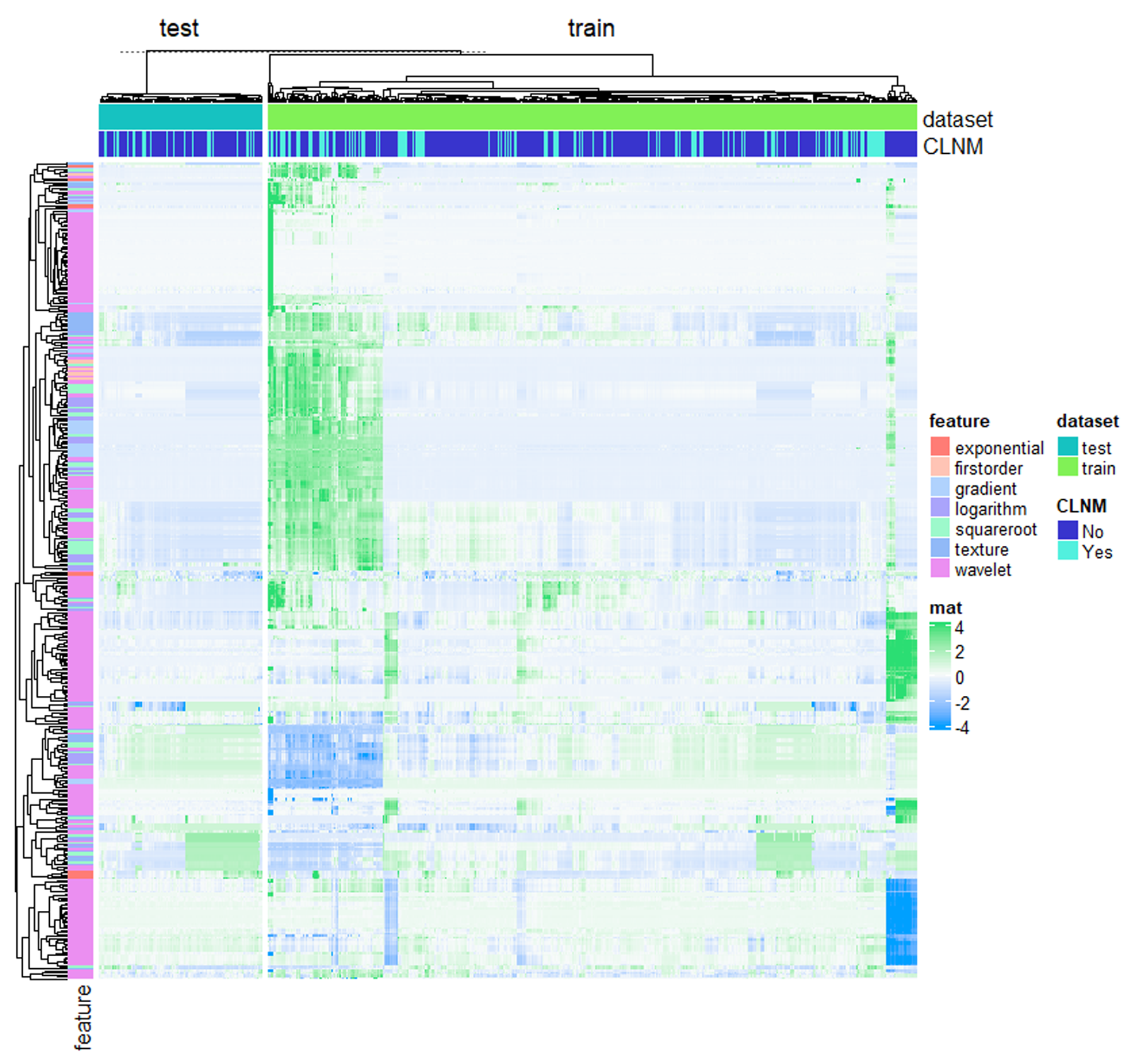

Supplement: Supplementary file 1 [file Presentation_1.zip › Appendix/Appendix Figures/Appendix Fig. 4 express heatmap.tiff]

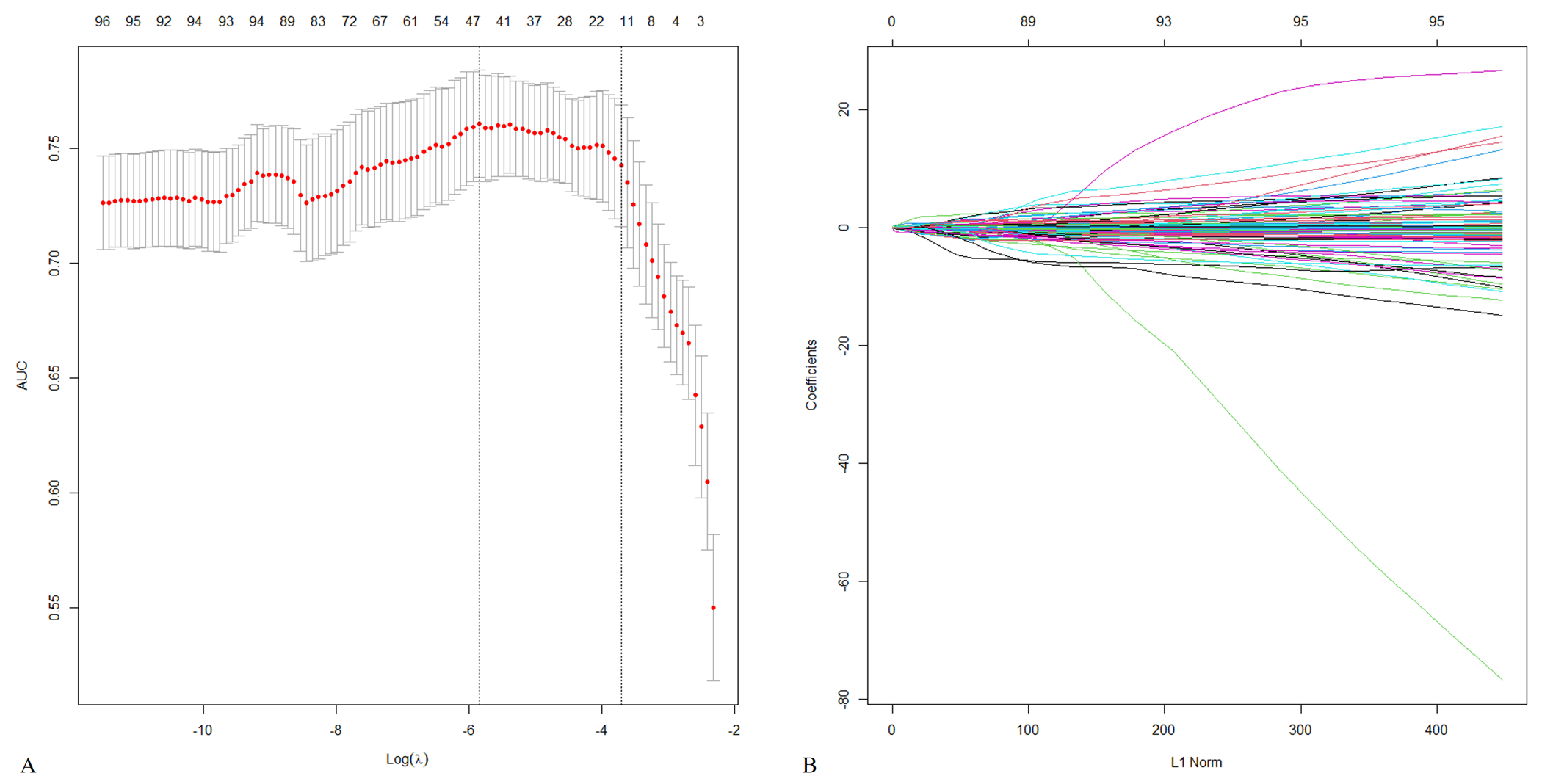

Supplement: Supplementary file 1 [file Presentation_1.zip › Appendix/Appendix Figures/Appendix Fig. 5.tif]

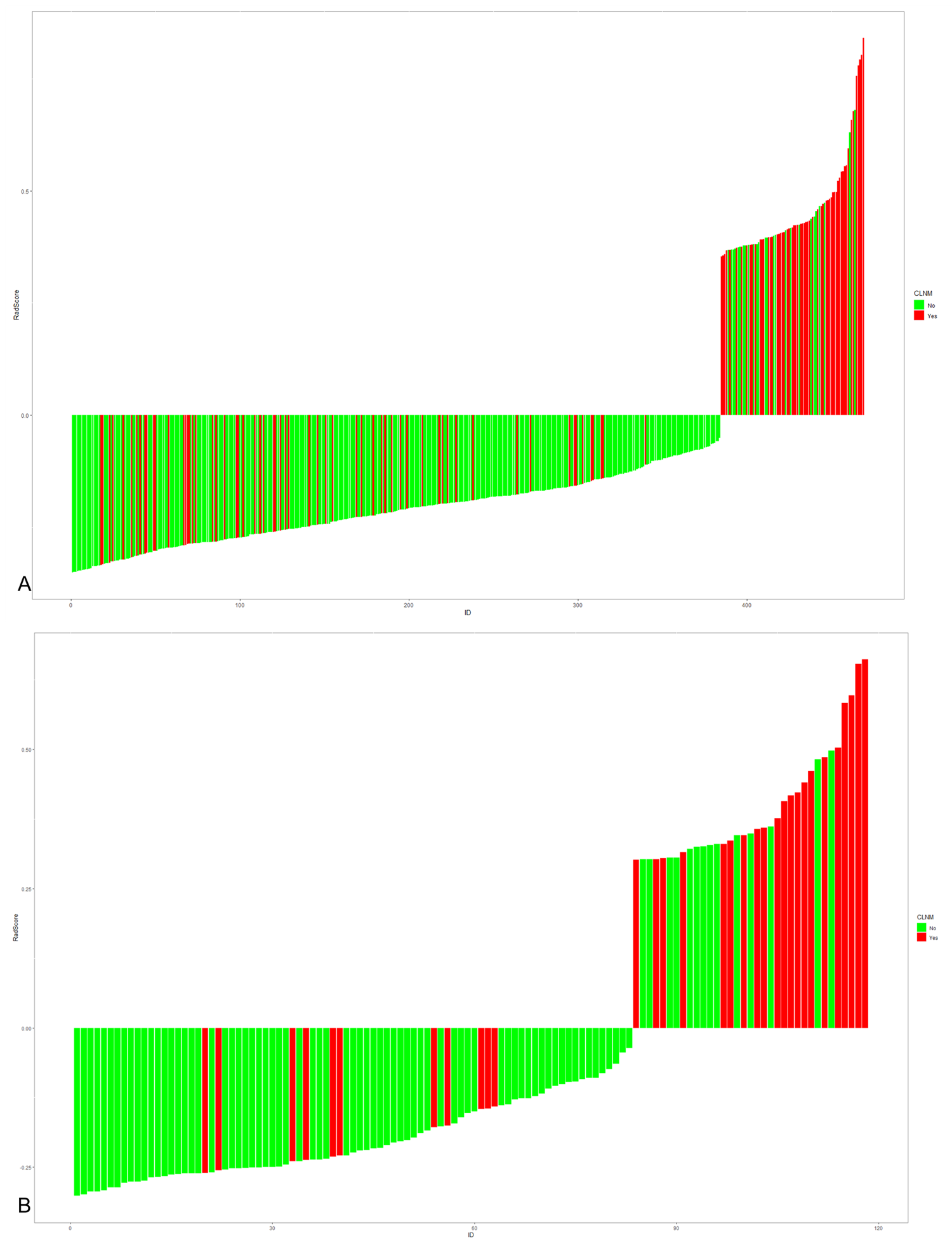

Supplement: Supplementary file 1 [file Presentation_1.zip › Appendix/Appendix Figures/Appendix Fig. 6.tif]

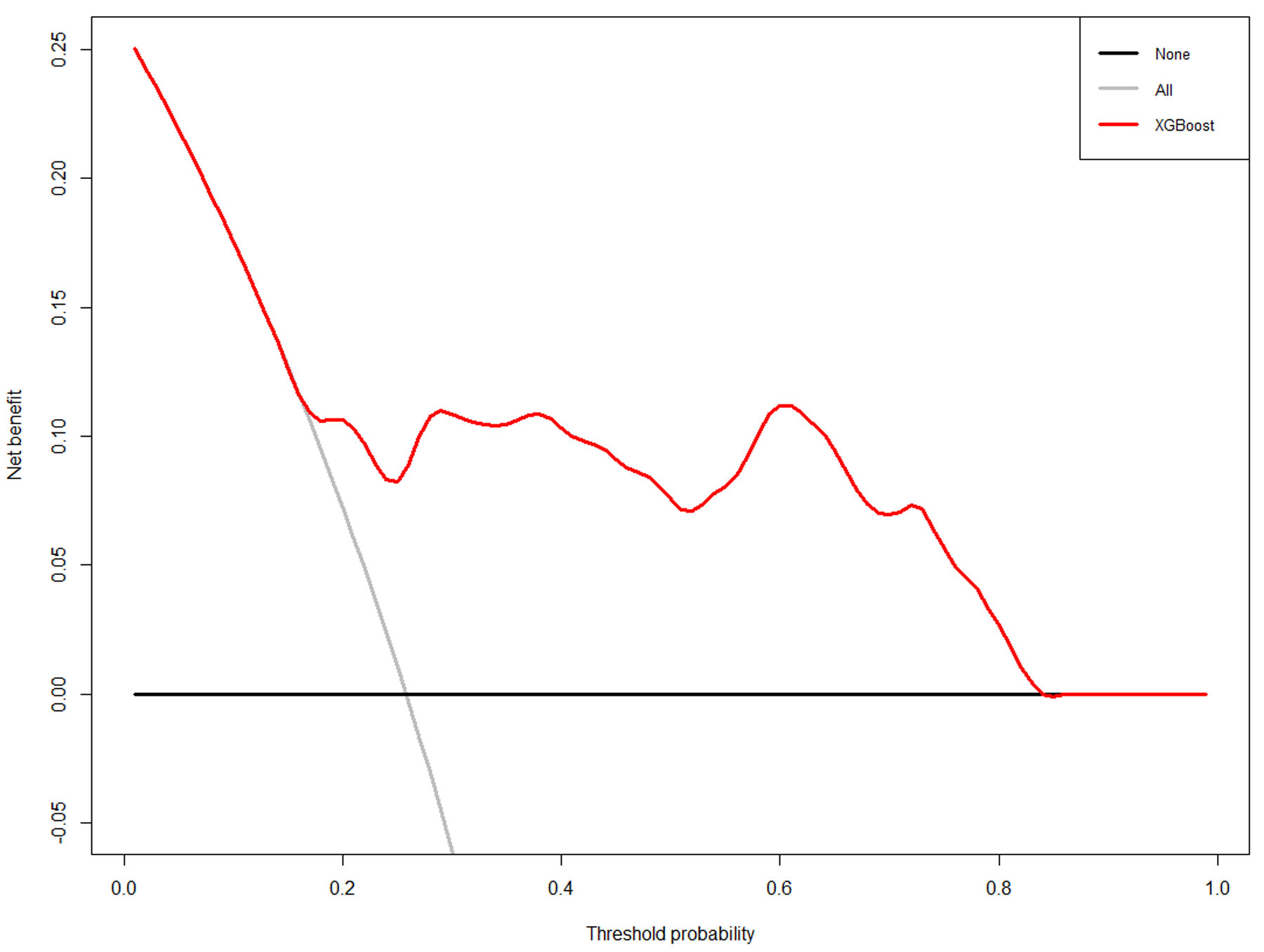

Supplement: Supplementary file 1 [file Presentation_1.zip › Appendix/Appendix Figures/Appendix Fig. 7 DCAPlot.tiff]

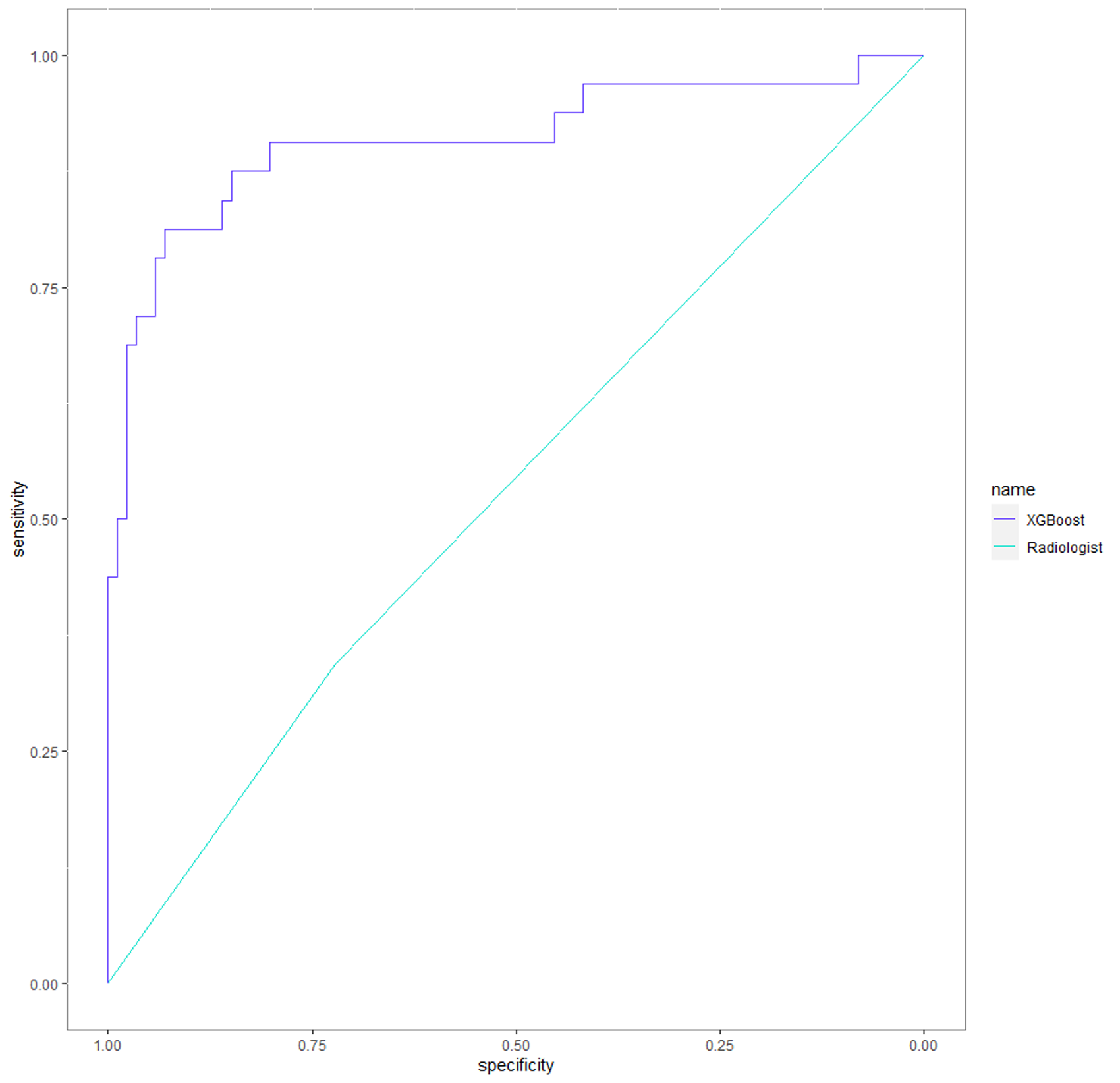

Supplement: Supplementary file 1 [file Presentation_1.zip › Appendix/Appendix Figures/Appendix Fig. 8 XGBooostVSradiology.tiff]

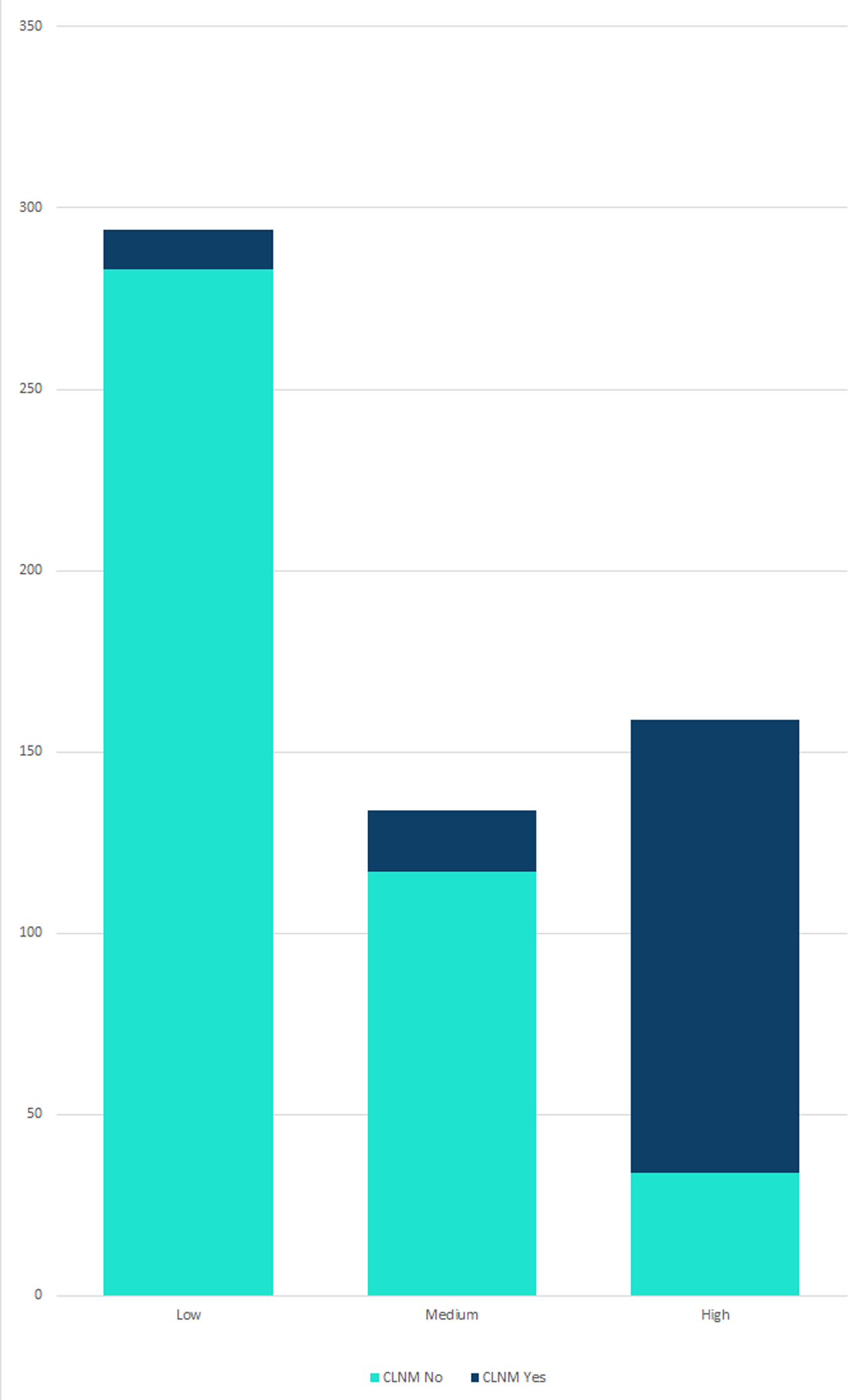

Supplement: Supplementary file 1 [file Presentation_1.zip › Appendix/Appendix Figures/Appendix Fig. 9 RiskBarPlot.tif]
